# A scoping review on metrics to quantify reproducibility: a multitude of questions leads to a multitude of metrics

**DOI:** 10.1098/rsos.242076

**Published:** 2025-07-15

**Authors:** Rachel Heyard, Samuel Pawel, Joris Frese, Bernhard Voelkl, Hanno Würbel, Sarah McCann, Leonhard Held, Kimberley E. Wever, Helena Hartmann, Louise Townsin, Stephanie Zellers

**Affiliations:** ^1^ Center for Reproducible Science, University of Zurich Institute of Epidemiology Biostatistics and Prevention, Zurich, Switzerland; ^2^ Department of Biostatistics, University of Zurich Institute of Epidemiology Biostatistics and Prevention, Zurich, Switzerland; ^3^ Department of Political and Social Sciences, European University Institute, Fiesole, Italy; ^4^ University of Bern, Bern, Switzerland; ^5^ Berlin Institute of Health at Charité QUEST Center for Responsible Research, Berlin, Germany; ^6^ Departmentof Anesthesiology, Pain and Palliative Care, Radboud University Medical Center, Nijmegen, Gelderland, The Netherlands; ^7^ Department of Neurology and Center for Translational, Neuro- and Behavioral Sciences, University Hospital Essen, Essen, Nordrhein-Westfalen, Germany; ^8^ Research and Innovation Office, Torrens University Australia, Adelaide, South Australia, Australia; ^9^ Institute for Molecular Medicine Finland, University of Helsinki, Helsinki, Uusimaa, Finland

**Keywords:** reproducibility, replicability, generalizability, translatability, meta-research, metrics

## Abstract

Reproducibility is recognized as essential to scientific progress and integrity. Replication studies and large-scale replication projects, aiming to quantify different aspects of reproducibility, have become more common. Since no standardized approach to measuring reproducibility exists, a diverse set of metrics has emerged and a comprehensive overview is needed. We conducted a scoping review to identify large-scale replication projects that used metrics and methodological papers that proposed or discussed metrics. The project list was compiled by the authors. For the methodological papers, we searched Scopus, MedLine, PsycINFO and EconLit. Records were screened in duplicate against pre-defined inclusion criteria. Demographic information on included records and information on reproducibility metrics used, suggested or discussed was extracted. We identified 49 large-scale projects and 97 methodological papers and extracted 50 metrics. The metrics were characterized based on type (formulas and/or statistical models, frameworks, graphical representations, studies and questionnaires, algorithms), input required and appropriate application scenarios. Each metric addresses a distinct question. Our review provides a comprehensive resource in the form of a ‘live’, interactive table for future replication teams and meta-researchers, offering support in how to select the most appropriate metrics that are aligned with research questions and project goals.

## 1. Introduction

Reproducibility of research results is often referred to as a cornerstone of science. Historically, the idea of replication as a means to establish the trustworthiness of a reported observation can be traced back at least 1000 years to the Persian scholars al-Biruni and al-Haytham [1]. Later, Galileo emphasized that he repeated his experiments on movement on the inclined plane a hundred times in order to give the results more credibility [2]. The first scientific society in modern Europe, the *Accademia del Cimento*, founded in Florence in 1657, considered replication to be such a fundamental concept that it chose *provando e riprovando* (to verify repeatedly) as the society’s motto. Similarly, the Royal Society of London declared replication of experiments as the sole method for establishing ‘matters of fact’ [1]. Yet, early authors were very vague regarding how they established that a replication confirmed the original observation. Even today, there is no universally accepted definition of ‘reproducibility’, as usage of the term and suggestions for how to establish or quantify reproducibility can vary widely among researchers and disciplines [3,4]. Acknowledging that there is an ongoing debate on the definition of different aspects of reproducibility, we will use the terms as suggested by the (iRISE) improving Reproducibility In SciencE consortium [5, p.6], for the purpose of our study. Here, replicability is defined as *the extent to which design, implementation, analysis, and reporting of a study enable a third party to repeat the study and assess its findings*, replication as *a study that repeats all or part of another study and allows researchers to compare their findings* and reproducibility as *the extent to which the results of a study agree with those of replication studies*. We refer to box 1 for a more detailed discussion of the terminology. The definition of reproducibility immediately asks for a specification of how to quantify the extent of agreement between a study and its replication. While there is no definition of reproducibility that is universally accepted across disciplines and research types, even less is known about the metric that best captures the reproducibility of a study or finding. However, selecting the most appropriate outcome for a reproducibility study^1^ is crucial to ensure the accuracy and credibility of research into the reproducibility of science.

Box 1: **On the definitions of reproducibility and replicability.**

The Oxford English Dictionary (OED [6]) defines *to reproduce* as to bring again into existence, to create from anew, to repeat in a more or less exact copy, or to give a specified quality or result when copied. *Reproducibility* is the capacity to be produced again, or the extent to which consistent results are obtained when produced repeatedly. The adjective *replicable*, on the other hand, is defined as able to be repeated experimentally, and the noun *replicability* is the property of being experimentally replicable. The use of *reproducibility* and *replicability* in the scientific literature has given rise to intense debates, as different and sometimes contradictory definitions have been put forward. Often, authors make no distinction and use both terms synonymously; others use the terms to distinguish whether a replication was done by the same or a new team, using the same or new data, or the same or new analysis [4,7–10]. We distinguish between two concepts.

*Reproducibility*: we define reproducibility as the extent to which the results of a study agree with those of replication studies [5]. This definition is inspired by the Federation of American Societies for Experimental Biology [11], defining reproducibility as the ability to achieve similar or nearly identical results using comparable materials and methodologies. Defined in this way, reproducibility is equivalent to what Goodman *et al*. [3] called *results reproducibility*. In research fields where researchers use existing data, reproducibility may be equivalent to computational reproducibility, as a full replication of a study is equivalent to re-running the analysis on the same data. In other fields, studies include both data generation or collection and analysis (e.g. intervention studies in medicine or psychology, experimental studies in life sciences, chemistry or physics). In such cases, a full or direct replication would require redoing the experimental part (data collection) and the analysis of newly collected data. A re-analysis of existing data would be considered a partial replication.

*Replicability*: a fundamental requirement of scientific studies—referred to as replication standard [12]—is that methods, study procedures and results must be described in sufficient detail and clarity that a third party could re-do the study and arrive at the same results (within uncertainty limits) without additional information from the author(s). To meet this requirement, it is not necessary that a replication is carried out, nor that it would produce the same result if it were. The reporting of the original study has to be done with sufficient detail, enabling the redoing of the study. A study that meets this replication standard can be considered replicable, and we therefore define replicability as ‘the extent to which design, implementation, analysis and reporting of a study enable a third party to repeat the study and assess its findings’ page 6 of [5].

The *strength* of these definitions lies in their broad applicability across fields and types of research, as well as their flexibility to assess only parts of a study. Earlier definitions, such as those presented in *The Turing Way* [10] or in Barba [4], were primarily developed for research drawing conclusions from quantitative data, potentially limiting their relevance for other disciplines or study types. Our definitions also allow for an important distinction between the replicability of the *research process* and the reproducibility of its *outcomes*.

An increasing number of articles has discussed the relevance of various metrics to define ‘successful replication’ in the pairwise comparison of original-replication study pairs. Hereafter, we define a successful replication as ‘a replication study for which the results agree with the corresponding original study’. ‘Agreement of results’ can mean different things: from an exact match of numeric values to matching conclusions. In a rapid review of replication studies in psychology published in 2013, Anderson & Maxwell [13] investigated the decision criteria for successful replication. They concluded that the majority of published replication studies (44 of the 50 included studies) classified the replication as successful when both studies came to the same conclusion based on statistical significance. Cobey *et al*. [14] conducted a scoping review of replication studies published in 2018 and 2019 in economics, education, psychology, health sciences and biomedicine to describe the epidemiological characteristics of this literature. They found large variability in how authors assessed reproducibility, although most of the included studies used a comparison of effect sizes to define success. Furthermore, large-scale reproducibility efforts, e.g. the replication projects in psychology [15], experimental economics [16] or cancer biology [17], all used a whole set of metrics based on statistical significance, effect sizes or methodology from meta-analysis to summarize the reproducibility of a research field. This list of traditional metrics for reproducibility includes the significance criterion, where a replication is considered successful if it finds a statistically significant effect in the same direction as the original study, and effect size comparisons, where success is determined by the similarity between the effect sizes of the replication and the original study. To investigate whether there is one best metric for the quantification of replication success, Muradchanian *et al*. [18] conducted a simulation study to examine the performance of a set of metrics in terms of their classification accuracy under varying degrees of publication bias. Their findings revealed no clear ‘winner’ across all simulation conditions, emphasizing that the choice of the most appropriate metric may depend on the specific context or objective of the analysis. In line with this, Anderson & Maxwell [13] directly link the criteria for replication success to distinct replication goals. Existing reviews examining the usefulness and limitations of various metrics for reproducibility (including Hung & Fithian [19] and Nosek *et al*. [20]) typically lack a systematic search of the literature. Moreover, they tend to focus on one narrow aspect of reproducibility and scenario of application: specifically, where a replication study applies the same design, methodology or analysis as the original study to newly collected data.

In our review, we aim to gain a more comprehensive overview of metrics that have been used or suggested to quantify, assess, explain or predict different types of reproducibility. We sought to identify all metrics used in larger studies and projects, as well as those suggested in methodological literature. To achieve this, we conducted a literature review of applied and methodological research. We did not restrict our comprehensive search to statistical metrics based on formulas, but rather included all papers where the authors claimed that they used or developed any type of measure to quantify or assess reproducibility or a related concept, regardless of their reproducibility definition. We addressed the following research questions: (i) which metrics have been used or suggested to quantify, assess, explain or predict reproducibility? and (ii) which of these metrics have solely been suggested theoretically, and which have been proposed or discussed together with information on their practical implementation (e.g. clear implementation steps, ready-to-use tools or open-source code)? We also identified the scenarios in which each metric proved most useful and associated each with a research question to guide users in interpreting the metrics. Additionally, we extracted details on any reported assumptions and limitations.

The metrics identified in our review are summarized in a table designed to inform various audiences in reproducibility research. A ‘live’ and interactive version of the table can be found on http://rachelheyard.com/reproducibility_metrics/. Target audiences include replication teams planning future reproducibility studies, newcomers to the field seeking a first comprehensive overview of available metrics and the broader meta-research community, particularly those requiring outcome measures for intervention studies aimed at improving reproducibility. Additionally, our findings will support peer reviewers and researchers alike in critically evaluating the appropriateness of metrics used in reproducibility efforts, ensuring they align with the study’s goal. This review is part of the work done by the iRISE consortium. iRISE is committed to mainstreaming equity, diversity and inclusion (EDI, see also https://osf.io/b4crd) and the iRISE glossary [5] contains definitions of EDI-related terms. Therefore, we collected data on potential content from included manuscripts that referenced any aspect of EDI, specifically with respect to the applicability or generalizability of the metrics and performed an exploratory analysis.

We first outline our review methods, including the paper eligibility criteria, search strategy, data screening and data extraction process in §2. The results are presented separately for the metrics used in large-scale reproducibility efforts (§3.3) and the metrics suggested in methodological research (§3.4). We finish with a discussion of our results, limitations and future directions in §4.

## 2. Methods

The protocol of the present study was preregistered on the Open Science Framework prior to initiating the literature screening and data extraction [21]. The protocol, as well as this manuscript, follows the PRISMA-ScR reporting guidelines for scoping reviews [22] (see the filled checklist https://osf.io/v7tas). Any deviations from the protocol were recorded and are discussed in §3.1. When referring to metrics, we include any metrics that provide a binary classifier of a study, part of a study or results of a study being reproducible. We also include any metrics that provide a continuous quantification of reproducibility or level of reproducibility (for example on a numeric scale, or from ‘not at all’ to ‘fully reproducible’) and are interested in any tools, algorithms or models that measure, aim at explaining or predicting reproducibility in a broader sense. Our search strategy was developed under the guidance of an information specialist (Robin Segerer, University Library Zurich) and aims to identify two classes of papers: *application papers* and *methodological papers*. Therefore, the review was divided into two parts:

(i) *application papers*—to gain an understanding of the metrics used to quantify, assess, explain or predict a specific type of reproducibility in practice, a list of large-scale reproducibility projects^2^ was compiled by the project team (available via our Zotero library^3^). They do not include single efforts to reproduce part or all of an original study. To qualify as a large-scale reproducibility project, the project team should, in addition to conducting the set of replication studies, attempt to summarize the results of the set of studies.
(ii) *methodological papers*—a systematic search was conducted to identify literature in which authors proposed or discussed metrics to quantify, assess, explain or predict any type of reproducibility.

The screening and data extraction of the application papers preceded and informed the screening and data extraction of the methodological papers.

### 2.1. Eligibility criteria

All papers, protocols or preprints discussing the methodology or the results of a large-scale reproducibility project were included as application papers. Such projects were defined as large-scale efforts to measure the reproducibility of a field, method, type of study or similar (e.g. [23,24]). These projects attempt to reproduce multiple previous results, to repeat a specific part of multiple previous studies or to repeat one analysis multiple times in independent teams. They further aimed at summarizing the results into a quantification of overall reproducibility. All manuscripts suggesting or discussing the use of specific metrics to quantify, assess, explain or predict a certain type of reproducibility were included as methodological papers. The aspect of reproducibility discussed in these papers had to align with the current definition of terms in the iRISE Reproducibility Glossary [5]. More specifically, papers using the same terminology but in a different, unrelated context (including translation in linguistics, image replication, sexual reproduction, cell or bacteria replications, virus reproduction ratio) were excluded. All years of publication and fields of research were included. For the systematic search of methodological papers, all languages were included, while the list of application papers was compiled by the project team and is therefore limited to English literature. Commentaries, editorials and opinion pieces were excluded unless it was apparent from the abstract that a metric or measure was suggested or discussed. Single study application papers, e.g. papers discussing single replications of single original findings, were excluded, because they generally used the same set of traditional metrics, including metrics based on statistical significance and effect size comparisons [14], and the effort of assessing such papers in depth was considered disproportionate to the amount of potential information to be gained.

### 2.2. Search strategy, information sources and screening

To collect the application papers, e.g. description of the methodology or the results of large-scale reproducibility efforts, two team members (R.H. and S.P.) initialized a list of projects that was complemented via a call for contributions (started beginning of December 2023, see https://osf.io/a2wrj). Once the list was finalized (mid March 2024), it was uploaded to the Systematic Review Facility (SyRF) [25], and five team members (H.H., J.F., L.T., R.H. and S.P.) screened the titles and abstracts of the documents for final inclusion. All documents were screened in duplicate, and conflicts were resolved by a third independent reviewer as automatically implemented in SyRF.

For the methodological papers, a systematic search was performed in the following databases: Scopus, MedLine (via Ebsco), PsycINFO (via Ebsco) and EconLit (via Ebsco), where the selection of discipline-specific databases was inspired by Cobey *et al*. [14]. The search strings can be found in appendix A. The literature search was performed on 13 May 2024. The search results were deduplicated in R (via their digital object identifier, DOI) and imported into SyRF. A screening guide was developed, see appendix B.1, tested and adapted using a random sample of 20 methodological papers. Six team members (H.H., J.F., L.T., R.H., S.P., S.Z.) screened titles and abstracts in duplicate and conflicts were resolved by a third independent reviewer as automatically implemented in SyRF. While screening, the reviewers had the option to annotate papers that were not, by definition, methodological papers, but documented an ‘interesting application’. A paper was labelled as an ‘interesting application paper’ whenever it was apparent from the title and/or abstract that the authors applied an innovative or non-traditional reproducibility metric (i.e. other than significance criterion, meta-analysis or effect size comparison).

During data extraction of the application papers and with the flagged ‘interesting application papers’, more potential methodological papers were retrieved. Additionally, a forward–backward reference and citation search was performed on the included methodological papers that were not flagged ‘interesting application papers’, via OpenAlex using the openalexR R package [26]. The titles of the papers identified via OpenAlex were subjected to a keyword search, and only those papers with at least one of the following terms in the title were retained for screening: *quantify, measure, evaluate, assess, quantifying, measuring, evaluating, assessing, metric, score, rating, quantification, measurement, evaluation* and *assessment*. The retained 296 potential methodological papers were pre-screened by one team member (R.H.). The records retained after pre-screening, as well as the potential methodological papers extracted from the application papers and the ‘interesting application papers’, were screened by four team members (J.F., L.T., R.H., S.Z.) using the screening guide in appendix B.2. Each document was screened in duplicate and conflicts were resolved by a third independent reviewer as automatically implemented in SyRF.

### 2.3. Data extraction

All data extraction was performed in SyRF. For the application papers, five team members (H.H., J.F., L.T., R.H., S.P.) extracted information on the research question or aim of the project, the type of project and, if applicable, the definition of reproducibility given by the authors, or inferred from the text. The type of project is of particular interest for application papers, as it determines what format of data is collected and what type of metrics can be used. McShane *et al*. [27] defined the types ‘many phenomena, one study’, where many original hypotheses are tested, each in one replication study, ‘one phenomenon, many studies’, where one original hypothesis is tested by many different teams or in many separate studies, and ‘many phenomena, many studies’, where many original hypotheses are tested in many separate studies. Information on the metrics to quantify reproducibility was extracted using a pre-defined list (with traditional reproducibility metrics such as ‘agreement in statistical significance’) and free text for less traditional metrics. If the authors mentioned other papers or documents with further information on the metrics used, their DOIs were retrieved and fed into the systematic search for methodological papers. Additionally, any text discussing limitations or assumptions related to the metrics used was extracted. Finally, text related to a discussion of EDI dimensions of the metrics was extracted (see §2.4 for more information). The full list of questions used for data extraction for the application papers can be found in appendix C.1. Each document was annotated by at least two reviewers. One team member (R.H.) merged the individual data extraction sheets together and reconciled any differences.

The ‘interesting application papers’ which were included in the screening of the methodological papers were annotated by four team members (H.H., L.T., R.H., S.Z.), notably to identify any potential methodological papers that were cited (the data extraction guide is in appendix C.2.1).

The 97 methodological papers were annotated by six team members (not in duplicate by H.H., J.F., L.T., R.H., S.P., S.Z.) using the extraction guide in appendix C.2.2. In particular, details on whether the metric was designed for the purpose of quantifying reproducibility, the particular type of reproducibility or related concept that the metric addresses and the type of measure, including a formula, a model or a metric derived from a study or survey (see the extraction guide for examples), were extracted. We also collected information on the implementation, the required data input, any assumptions or limitations discussed, as well as mentions of EDI dimensions.

After all the information was extracted on a paper level, one team member (R.H.) identified the distinct metrics that were either used in the application papers or suggested and discussed in the methodological papers, and composed a table on the level of reproducibility metric. This table was reviewed by the other team members.

### 2.4. Exploratory analysis on equity, diversity and inclusion dimensions considered in reproducibility assessment

Since data on how dimensions related to EDI are considered in the reproducibility space is limited, it is of great value to collect EDI-relevant data whenever possible. We therefore collected any mention of EDI in the included records, with a focus on epistemic diversity, defined in the iRISE glossary [5]. We were specifically interested in whether authors who suggested or used a certain reproducibility metric discussed its applicability or generalizability across research fields, research types or research communities. The extracted EDI content was reviewed by R.H. and S.Z. and grouped into topics for descriptive purposes, based on the EDI terms in the iRISE glossary [5]. This analysis was purely exploratory and not preregistered.

## 3. Results

### 3.1. Protocol amendments

The search string for the methodological papers was adapted to be more specific and ensure the number of records to screen was feasible for our small team. To narrow the scope of our manuscript, review questions 3 and 4 from the protocol on the interpretation, assumptions and limitations were only answered in a descriptive manner, based on the limited information extracted from the included records. A more focused discussion on the interpretation, assumptions and limitations of each metric remains to be performed. We decided against using Rayyan [28] for screening and instead performed both the screening and data extraction in SyRF. While the forward–backward search of the references and citations was mentioned in the protocol, the exact procedure was not pre-specified. We added an exploratory data analysis on the EDI dimensions.

### 3.2. Included records

**Figure 1.**
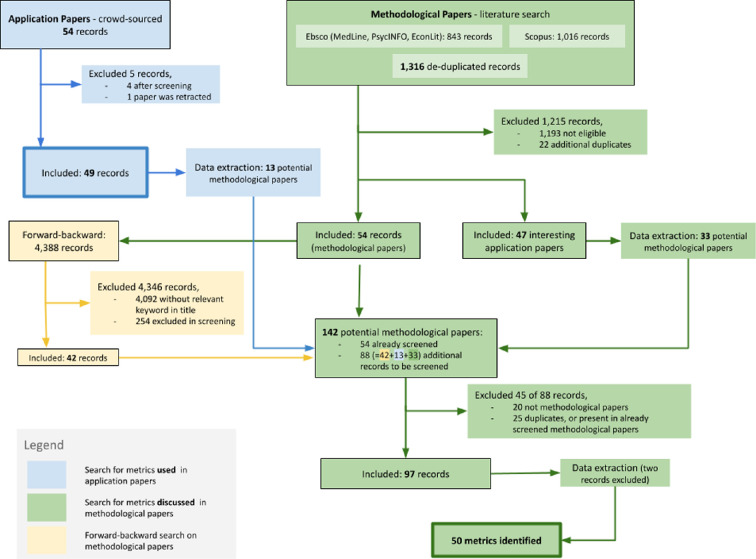
Flow chart of the search strategy for both application and methodological papers.

As outlined in figure 1, our research team identified 54 records potentially discussing a large-scale reproducibility effort. Following screening, 50 of these papers were retained for data extraction, while one was later retracted by the journal and therefore excluded from our analysis. During data extraction of the 49 included application papers, we identified 13 potential methodological papers. The literature search for the methodological papers yielded 1316 records, of which 1215 were excluded during the screening process. We retained 101 records, of which 47 were flagged as ‘interesting application papers’. The remaining 54 records were classified as methodological papers. Data extraction from the ‘interesting application papers’ led to the identification of an additional 33 potential methodological papers. Subsequently, a forward and backward citation search on the 54 included methodological papers resulted in 4346 records, with 296 of these containing relevant keywords in their title. After screening the 296 records, 42 more records were added to the list of potential methodological papers. In the final step, we screened the 88 potential methodological papers, identified through data extraction and forward and backward citation search, after deduplication. After data extraction of the methodological papers, one record was excluded as it was written in Czech, and no team member was fluent in Czech. We also found one more duplicate. Ultimately, a total of 95 distinct methodological papers were included in this review. In the following sections, the results for the application papers and methodological papers will be presented separately.

### 3.3. Application papers

#### 3.3.1. Characteristics of the included application papers

Table 1 gives a first impression of the characteristics of the 49 included application papers. Most large-scale reproducibility efforts were performed in the social sciences (67%) and only a minority in the health and life sciences (20%) and physical sciences (12%). Less than half of the included records (23 out of 49 = 47%) clearly defined what they meant by ‘reproducibility’, i.e. we were able to identify a clear definition in the paper. When categorizing the aspect of reproducibility using the texts, we concluded that most records (27 out of 49 = 55%) report that in their effort, they used the same analysis on different data, defined as a form of ‘replication’ in Voelkl *et al*. [5]. Among the included records, we found an equal share of project types. One of the project records presented two types: the protocol by Page *et al*. [29] presents the REPRISE project, a large effort encompassing four studies, where studies two and three were of interest in our review; one was classified as a ‘many phenomena, many studies’ project and one a ‘many phenomena, one study’ project. Most of the included reproducibility efforts were conducted by a large team of authors (median number of project authors = 24), while some were conducted by only one or a handful of authors. The included papers were fairly recently published (median year of publication = 2020), and were generally heavily cited (median number of citations = 61, 28 September 2024).

**Table 1.** Characteristics of the included application papers.

	*n* (%), unless otherwise indicated
total records	49
**field of research (OpenAlex)**	
health and life sciences	10 (20.4%)
physical sciences	6 (12.2%)
social sciences	33 (67.3%)
**authors defined reproducibility?**	
no	26 (53.1%)
yes	23 (46.9%)
**aspect of reproducibility**	
combination^a^	2 (4.1%)
different data—different analysis	3 (6.1%)
different data—same analysis	27 (55.1%)
same data—different analysis	13 (26.5%)
same data—same analysis	4 (8.2%)
**type of project**	
many phenomena, many studies	16 (32.7%)
many phenomena, many studies; many phenomena, one study	1 (2%)
many phenomena, one study	15 (30.6%)
one phenomenon, many studies	17 (34.7%)
**number project authors**	
median	24
range	1–260
**citation count (extracted via openalexR [26] on 28 September 2024)**	
median	61
range	0–6739
**year of publication**	
median	2020
range	2007–2024
**number of measures used**	
median	2
range	1–12
**agreement in statistical significance**	
no	17 (34.7%)
yes	32 (65.3%)
**agreement in effect size**	
no	14 (28.6%)
yes	35 (71.4%)
**meta-analysis of study results**	
no	40 (81.6%)
yes	9 (18.4%)
**subjective assessment**	
no	35 (71.4%)
yes	14 (28.6%)
**used none of the pre-defined measures**	
no	45 (91.8%)
yes	4 (8.2%)

^a^ These papers presented projects with several sub-projects looking at different aspects of reproducibility.

#### 3.3.2. Characteristics of reproducibility metrics used

**Figure 2.**
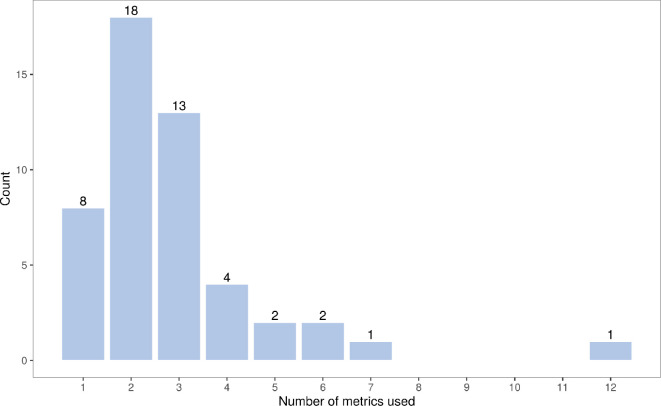
The total number of metrics used in the application papers to summarize reproducibility.

Eight (16%) reproducibility efforts used only a single metric, while the remainder used at least two metrics to evaluate reproducibility (figure 2). A total of 12 metrics were recorded for Wang *et al*. [30]. The metrics used were of varying types and investigated agreement in significance or effect size, using meta-analysis methodology or subjective assessment.

**Figure 3.**
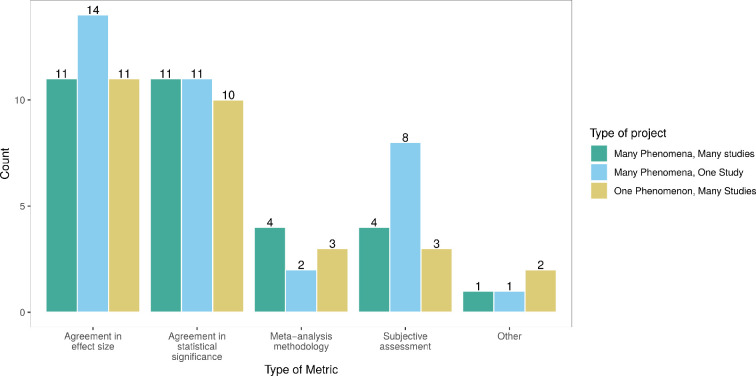
Count of mentions of different types of metrics depending on the type of project. Note that projects classified as more than one (combined) type were split into multiple projects.

**Figure 4.**
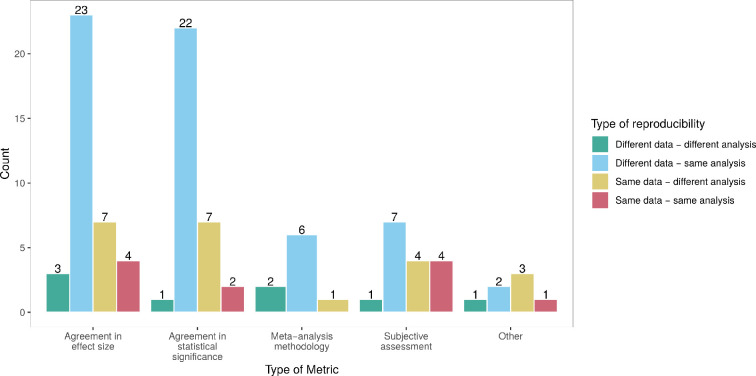
Count of mentions of different types of metrics by type of reproducibility. Note that projects classified as investigating several types of reproducibility were split into multiple projects.

*Agreement in statistical significance*: thirty-two (65%) of the included application papers used at least one metric based on statistical significance. These 32 projects were equally likely to be either type of project, as shown in figures 3 and 4, which shows that most of these projects repeated the same analysis on different data. Usually, ‘many phenomena, one study’ project types, like Errington *et al*. [17], investigate whether the original and replication studies found a significant effect in the same direction. For ‘one phenomenon, many studies’ or ‘many phenomena, many studies’ projects like Klein *et al*. [31], measuring reproducibility based on statistical significance means computing a proportion of samples or replications that rejected the null hypothesis in the expected direction. ‘Many phenomena, many studies’ project types, including the Brazilian Reproducibility Initiative [32], where each study was replicated three times, usually employed a pooled version of the effect sizes of the replication studies to assess reproducibility. ‘One phenomenon, many studies’ project types, on the other hand, reported rates, shares or counts of studies or analyses obtaining statistically significant results, as for example, Schweinsberg *et al*. [33].

*Agreement in effect size*: seventy-one per cent (35 out of 49 = 71%) of the application papers used at least one metric based on the agreement in effect sizes. These metrics come in different forms. Irvine *et al*. [34] informally describe how the original and replication effect sizes compare to each other in tables and figures. One of the seven reproducibility metrics used by Errington *et al*. [17] was to simply check that the direction of the effect was the same in the original and replication studies. Cova *et al*. [23] and Camerer *et al*. [16] used a binary measure assessing whether the 95% confidence interval (CI) of the replication effect size includes the original effect size. Since this metric does not acknowledge sampling error in both the original and the replication study, Camerer *et al*. [16] and Boyce *et al*. [35] investigated whether the replication effect sizes were included in a 95% prediction interval of the original effect size, as suggested by Patil *et al*. [36]. For projects where multiple replication studies were performed for one phenomenon or original study, the effects for all replications were aggregated and then compared with the original effect (as in Ebersole *et al*. [37]). Klein *et al*. [31], a ‘many phenomena, many studies’ project, investigated variation across samples and settings using intra-class correlation coefficients and the heterogeneity of effect sizes using Cochran’s Q and $I^{2}$. Chang *et al*. [38], who followed Wang *et al*. [39] to design their project, assessed reproducibility using standardized differences to investigate whether the effect sizes of original and replication studies (here randomized controlled trials versus real-world evidence emulations) were significantly different. In addition, they claimed successful replication (or emulation) if the effect estimates of the replication fell within the 95% CI of the original study. Ebersole *et al*. [40], Errington *et al*. [17] and Boyce *et al*. [35] used *p-original*, defined as the *p*-value for the null-hypothesis that the effect size of the original study and the effect size of the replication study (or effect sizes of several replication studies) follow the same distribution [41]. This metric can take effect size heterogeneity into account and assess statistical consistency between original and replication studies.

*Meta-analysis of study results*: only nine (18%) of the included application papers reported that they used a meta-analysis of study results to decide on successful replication or degree of reproducibility. In ‘many phenomena, one study’ projects, this usually entailed performing a fixed-effect meta-analysis of the findings from the original and the corresponding replication study and flagging successful replication if the meta-analytical effect size was found to be significant in the same direction as the original effect (as in [15–17,32]) The remaining reproducibility projects, specifically ‘many phenomena, many studies’ and ‘one phenomenon, many studies’, performed meta-analyses, usually random-effects, of all replication effect sizes to assess and quantify reproducibility (e.g. [40,42–44]). If there was an original study, these meta-analytical results were then compared with the original results. Ebersole *et al*. [40] used meta-analytical approaches to investigate whether certain interventions could improve reproducibility.

*Subjective assessment*: twenty-nine per cent (14 out of 49 = 29%) of the application papers reported using some form of subjective or narrative assessment of reproducibility. This often implied asking replication teams, informally or using a survey questionnaire, for their assessment on the reproducibility of a study after having performed its replication [15,23,34,45]. More specifically, the replication team in Naudet *et al*. [46], for instance, classified papers into four categories: ‘fully reproduced’, ‘not fully reproduced but same conclusion’, ‘not reproduced and different conclusion’ and ‘not reproduced (or partially reproduced) because of missing information’. Boyce *et al*. [35] used a subjective replication score coded on a scale from [0, 0.25, 0.5, 0.75], which allowed raters to subjectively summarize multiple important outcomes or features of reproducibility. Low *et al*. [47] summarized the methodology used and conclusions drawn from two independent systematic reviews in a narrative manner. Other projects used so-called ‘prediction markets’, in which experts trade contracts on the possible outcome of the replication study, informed by the results of an original study and information on the design of a planned replication study (among others [16,48]). The market price can then be interpreted as the predicted reproducibility of the study. Alipourfard *et al*. [49] explain that they will use the repliCATS platform [50], which uses a modified form of a Delphi protocol to aggregate expert reproducibility assessments. In their project, where two datasets were re-analysed by four research teams, using either Bayesian or frequentist statistics, Dongen *et al*. [51] summarized the findings only in a subjective and narrative manner during discussions. The RepliSims project presented in Luijken *et al*. [52] describes the differences in the results of simulation studies in a qualitative and narrative way: ‘are trends in the results moving in the same direction or do the performance rankings of different simulation scenarios match those in the original study?’

*Additional metrics and analyses*: in addition to the metrics described above, some application papers used less traditional metrics to summarize the reproducibility of findings. Often, these were secondary or complementary analyses of the results. Specifically, Milcu *et al*. [43, p.282], a ‘one phenomenon, many studies’ project, used Tukey’s post-hoc honest significant difference test [53], to investigate ‘how many laboratories produced results that were statistically indistinguishable from one another’. Schweinsberg *et al*. [33], who asked several teams of analysts to answer the same research question, examined whether independent analysts would arrive at similar analyses and statistical results, and performed a multiverse analysis using the Boba approach as suggested in Liu *et al*. [54]. The Boba multiverse gave the project authors an opportunity to further understand which analysis choices played a major role in creating differences in the independent analysts’ results. In the Yale Open Data Access Medtronic Project (Low *et al*. [47]), two independent research teams used the same data and analysis, and the project authors not only compared the final results and conclusions of the two teams but also were particularly interested in differences in inclusion criteria and statistical methodology applied on the data, which were summarized in a narrative fashion. Many replication projects summarized differences in original and/or replication studies in a descriptive manner, including percentages, counts or number of differences and correlation coefficients (e.g. [44,55,56]). Bastiaansen *et al*. [57] and Huntington-Klein *et al*. [55], for example, recorded differences in processing and analysis steps and decisions. Wang *et al*. [30] used calibration and Bland–Altman plots to represent their findings and assess agreement of original and replication results.

#### 3.3.3. Limitations and assumptions of metrics discussed in application papers

For less than one-third (15 out of 49 = 30%) of the included application papers, we extracted discussions on assumptions or limitations related to specific metrics or measures to summarize or investigate reproducibility. Milcu *et al*. [43, p.285], for example, mentioned that using statistical significance to determine reproducibility might be ‘viewed as overly restrictive’. They argue that they used this approach owing to the lack of a better alternative. Cova *et al*. [23, p.16] mentioned that the use of statistical significance as a replication success criterion for original ‘null’ results is ‘especially dubious’, which was recently discussed in Pawel *et al*. [58]. Some reproducibility projects reported that they are specifically using subjective assessment metrics because they accommodate the consideration of multiple outcomes of interest and are applicable across a diverse set of outcome measures [35], while others mention the subjectivity as a limitation [45]. Wang *et al*. [30] discuss that the proportion of studies with effect estimates of the same sign is imperfect as a metric for studies with small effect sizes, as the smallest implementation differences could result in a sign change in the reproduction attempt. In the next section, some of the many of the metrics used in application projects are explained in more detail.

### 3.4. Methodological papers

#### 3.4.1. Characteristics of the included methodological papers

Of the 95 distinct records for which data were extracted, more than half (57 out of 95 = 60%) were categorized in the field of social sciences by openalexR. Sixty per cent (57 out of 95 = 60%) were original research papers, 17% (16 out of 95 = 17%) were review papers and 15% (14 out of 95 = 15%) were classified as tutorial papers (table 2). We extracted a total of 50 distinct reproducibility metrics from these records. Table 3 summarizes the key attributes of the metrics. Note that all metrics used in the application papers were included, except for the Boba multiverse approach used in Schweinsberg *et al*. [33], and the comparison of study results using various descriptive statistics, because those methods are less suited for the quantification or classification of reproducibility.

**Table 2.** Summary of methodological papers included.

	*n* (%)
total records	95
**field of research**	
health and life sciences	11 (11.6%)
physical sciences	27 (28.4%)
social sciences	57 (60%)
**type of paper**	
conference paper	1 (1.1%)
editorial, comment or similar	7 (7.4%)
original research paper	57 (60%)
review paper	16 (16.8%)
tutorial paper	14 (14.7%)

**Table 3.** Summary statistics of attributes of identified reproducibility metrics.

	*n* (%)
**total number of metrics**	50
**designed for reproducibility**	
no^a^	20 (40%)
yes	30 (60%)
**type of reproducibility**	
different data—different analysis	1 (2%)
different data—same analysis	27 (54%)
different data—same/different analysis	4 (8%)
same data—different analysis	1 (2%)
same data—same analysis	1 (2%)
same data—same/different analysis	1 (2%)
same/different data—same analysis	5 (10%)
same/different data—same/different analysis	10 (20%)
**type of metric**	
a formula and/or statistical model	37 (74%)
a framework	3 (6%)
a graph	3 (6%)
a study, survey or questionnaire	4 (8%)
an algorithm	3 (6%)
**purpose of metric**	
to classify	3 (6%)
to quantify	16 (32%)
to quantify and classify	21 (42%)
to quantify and explain	4 (8%)
to quantify and predict	6 (12%)
**type of assessment**	
qualitative	3 (6%)
qualitative and quantitative	5 (10%)
quantitative	42 (84%)
**implementation**	
clear implementation	1 (2%)
easy to implement	13 (26%)
hard, costly or unclear implementation	11 (22%)
ready-to-use closed tool provided	1 (2%)
ready-to-use open tool provided	24 (48%)
**data input**	
original raw data, code and/or software	3 (6%)
qualitative data, surveys or questionnaires	3 (6%)
results—figures	1 (2%)
results—figures, numbers and tables	2 (4%)
results—number and tables	37 (74%)
text, meta-data and information on design	4 (8%)

^a^ Includes unclear.

#### 3.4.2. Characteristics of the identified reproducibility metrics

Sixty per cent (30 out of 50 = 60%) of the metrics were specifically designed to assess reproducibility or a closely related concept, while the remaining 40% (20 out of 50 = 40%) were initially proposed for a different context, but used or suggested to be used in reproducibility studies. We extracted 37 metrics (37 out of 50 = 74%) that were formulas or statistical models. A type of metric we did not expect to find was ‘a framework’. Note that we did not pre-define this type nor what a ‘framework’ is, but instead adopted it from the methodological papers that first discussed these metrics. They either formalize conditions or outline a standardized workflow to quantify or interpret reproducibility. Four metrics summarize the reproducibility in a graphical representation, while another four quantify reproducibility using a study, a survey or a questionnaire. Three metrics are based on an algorithm. The ‘purpose of metric’ informs on whether the metric quantifies reproducibility in a continuous way or classifies it into ‘reproducible’ versus ‘not reproducible’ or replication success versus failure. Some metrics were specifically presented as being useful to explain or predict reproducibility. Most of the metrics (47 out of 50 = 94%) can be used to quantify reproducibility in a continuous manner. Twenty-four (24 out of 50 = 48%) were proposed or discussed together with a ready-to-use open tool or open-source software and code, while 11 metrics (11 out of 50 = 22%) were classified as hard or costly to implement. This was mostly owing to the metric relying on costly data retrieved using a study, e.g. prediction markets, or because the implementation was not clearly described. A large majority (39 out of 50 = 78%) use results in the form of numbers and tables to quantify or assess reproducibility.

Table 4 presents the descriptions of the 50 identified metrics, including their name, a brief description, the research questions they address, application scenarios, their purposes and relevant references (when they were first mentioned, discussed or applied in the context of reproducibility). The metrics are organized by type: first, the 37 metrics that are based on formulas and statistical models, followed by those using frameworks, graphs and studies, surveys or questionnaires. A more detailed version of the table, including information on their implementation, data input requirements, the extracted assumptions and limitations, is available online (http://rachelhey.github.io/reproducibility_metrics/). The assumptions and limitations listed are drawn directly from the reviewed records. All identified metrics come with some assumption or limitation, and each targets a specific research question. Thus, there is no single ‘best’ metric to quantify, classify, explain or predict reproducibility in general. Replication teams and meta-researchers should first define the research question they seek to answer and then select the most suitable metric and project type. In the following sections, we first summarize ‘statistical metrics’ (i.e. metrics based on formulas and statistical models), followed by a discussion of the other types of identified metrics.

**Table 4.** Metrics table: summary of the 50 identified metrics, ordered alphabetically and grouped by the type of metric: a formula or statistical model, a framework, a graph, a study, survey or questionnaire, or an algorithm. (The name and description of the metric is followed by one or several research questions summarizing the type of question the metric can answer. The scenario of application gives insights into the type of project design needed to compute or use the metric. We then collapsed all the references for further reading, where have the metrics first been mentioned in relation to reproducibility, which papers discussed them further and which application papers demonstrate how to use them.)

name (also called/related to)	description	research question	scenario of application	purpose of metric	references
**a formula and/or statistical model**					
Bayes factor: equality-of-effect-size BF test	this test compares the null hypothesis that the effect sizes from two experiments (o and r for original and replication) are equal against an alternative hypothesis that they are not. Suppose $H_{0}:\theta_{o}=\theta_{r}$ and $H_{1}:\theta_{o}\neq \theta_{r}$, then the equality-of-effect-size Bayes factor is defined as $B_{01}=\frac{f(Y_{o},Y_{r}\;|\;H_{0})}{f(Y_{o},Y_{r}\;|\;H_{1})},$ where $f(Y_{o},Y_{r}|H_{i})$ is the marginal likelihood of the data under hypothesis $H_{i}$ with i∈{0,1}. $B_{01}$ higher than 1 indicate support for $H_{0}$ and is indicative of a successful replication	‘what is the evidence for the effect size in the replication attempt being equal versus unequal to the effect size in the original study?’	two exchangeable studies: one original and one replication	to quantify	first mentioned in [59]. Discussed in [60,61]
Bayes factor: fixed-effect meta-analysis BF test (meta-analytic BF)	the meta-analytic Bayes factor quantifies the evidence provided by the data of several experiments/studies for the hypothesis that the true effect is present ($H_{1}$) versus absent ($H_{0}$): $B_{10}=\frac{f(Y_{1},...,Y_{M}\;|\;H_{1})}{f(Y_{1},...Y_{M}\;|\;H_{0})},$ where $f(\ldots |H_{i})$ is the marginal likelihood of the data under hypothesis $H_{i}$ with i∈{0,1}. A high $B_{10}$ indicates that the evidence from the pooled data supports $H_{1}$.	‘when pooling all data, what is the evidence for the effect being present versus absent?’	a series of exchangeable studies: one original and many replications; many replications without an original	to quantify	first mentioned in [62]. Discussed in [18,60,61]. Used in [44]
Bayes factor: independent Jeffreys–Zellner–Siow BF test (default BF)	this test compares the null hypothesis that the effect size is zero against an alternative hypothesis that the effect is not zero. Suppose $H_{0}:\theta =0$ and $H_{1}:\theta ∼\text{Cauchy}(0,1)$, then the Bayes factor is defined as: $$B_{10}=\frac{f(Y\;|\;H_{1})}{f(Y\;|\;H_{0})},$$ where $f(Y|H_{i})$ is the marginal likelihood of the data Y under hypothesis $H_{i}$ with i∈{0,1}. $B_{10}$ higher than 1 indicate support for $H_{1}$, whereas lower than 1 indicate support for $H_{0}$. In the replication setting, the Bayes factor is used to test the absence or presence of an effect in the replication study. Note that the Jeffreys–Zellner–Siow prior is a prior that is specifically designed for the *t*‐test/linear regression setting (normal data with unknown mean and variance)	‘what is the evidence for the effect being present or absent in light of a replication attempt, given that we know relatively little about the expected effect size beforehand?’	two exchangeable studies: one original and one replication	to quantify	discussed in [18,60,61,63]. Used in [64]
Bayesian evidence synthesis (variant: meta-analysis model-based assessment of replicability (MAMBA))	the approach assumes that multiple studies exist which investigate a common general theory. These studies might be so diverse in design and measurements, that the study-specific informative hypotheses reflecting the common theory can differ. First, the evidence for or against the hypothesis of interest in each individual study is quantified. The evidence is then pooled over studies, providing a joint level of support for the general theory. The aggregation uses updated model probabilities, that is, the posterior odds after observing a first dataset are used as the prior odds for the second study; and the posterior odds after inclusion of the second study are used as the prior odds for the third study. This process can be repeated for each additional replication study, as presented in $${\left(\frac{P(H_{1}\;|\;Y)}{P(H_{2}\;|\;Y)}\right)}^{N}=\frac{P(H_{1})}{P(H_{2})}\prod_{n=1}^{N}(B_{12}{)}^{n},$$ where n=1,…,N indicates the number of studies and Y denotes the data. Note that the prior odds before the first study $P(H_{1})/P(H_{2})$ is often set to one, reflecting no preference for either hypothesis before any data was observed. A closely linked variant of this is the MAMBA, introduced for replicability for genome data	‘given several conceptual replications with substantial diversity in data, design and methods but investigating the same theory, what is the evidence underlying a certain theory of interest?’	several substantially different replications investigating the same theory of interest	to quantify	first mentioned in [65]. Discussed in variant for genome data in [66]
Bayesian mixture model for reproducibility rate	it is a model for the p-values from the original results and the replications, in order to assess the reproducibility rate and to investigate whether some characteristics of the studies are associated with how likely they reproduce. In the mixture model, each pair of p-values (original and replication) comes from a mixture distribution where one component describes the p-value behaviour under the null hypothesis and the second under the alternative. All included original studies claim a significant result, the weight given to the second component of the mixture can be seen as a reproducibility rate. As such, the model is linked to the significance criterion	‘given the results (p-values) from a set of original and replication studies, what is the rate of reproducibility, and how is it related to certain aspects of the experiments?’	several pairs of original and replication studies	to quantify and explain	first mentioned in [67]
confidence interval: original effect in replication 95% CI (coverage)	for an original-replication study pair, this metric entails a binary check on whether the original effect size is included in the 95% confidence interval of the replication effect size. When several original-replication study pairs are considered, coverage is calculated as the proportion of pairs in which the original effect was in the CI of the replication	‘given an original effect size, (what is the probability that) does a repetition of the experiment, with an independent sample of participants, produce(s) a CI that overlaps with the original effect?’	one original and one replication study; or one original and many replication studies	to quantify and classify	first mentioned in [68]. Discussed in [69,70]. Used in [15–17,23,64]
confidence interval: replication effect in original 95% CI (capture probability)	for an original-replication study pair, this metric entails a binary check on whether the replication effect size is included in the 95% confidence interval of the original effect size. When several replication studies are performed, the shares of replications in that interval is captured via the capture probability, which is defined as the percentage of replication means, that (will) fall within a given original CI	‘given an effect size and 95% CI, (what is the probability that) does a repetition of the experiment, with an independent sample of participants, give(s) an effect that falls within the original CI?’	one original and one replication study; or one original and many replication studies	to quantify and classify	first mentioned in [68]. Discussed in [63,71]. Used in [17,38,39]
consistency of original with replications, $P_{\text{orig}}$	this metric is defined as the *p*-value for a null-hypothesis that the effect size of the original study and the effect size of the replication study (or effect sizes of several replication studies) follow the same distribution	‘to what extent are the replication effect sizes consistent with the effect size of an original study?’	one original study and several replication studies	to quantify	first mentioned in [72]. Discussed in [41,73]. Used in [35,40]
continuously cumulating meta-analytic approach	continuously cumulating meta-analysis (CCMA) uses standard meta-analytic calculations in a continuing fashion after each new replication attempt completes. Instead of simply noting whether each individual replication attempt reached significance, CCMA combines the data from all studies that were completed so far and computes meta-analytic indexes to quantify the evidence	‘given subsequent replications that were performed to date, what is the current evidence for an effect?’	one original study and several replication studies; or several replications	to quantify	first mentioned in [74]. Discussed in [13,69,75]
correlation between effects	replication is assessed in terms of the linear relationship between effect estimates, including numerically with the Pearson or Spearman correlation as well as visually with scatterplots. For successful replications the correlation should be close to 1	‘do the replication studies and the original studies produce effects that are correlated?’	several pairs of original and replication studies	to quantify	discussed in [76]. Used in Wang *et al*. [30]
correspondence test	this measure combines a difference (related to the *Q*-test) and equivalence test in the same framework. The correspondence test allows for a more nuanced inference regarding replication success or failure based on whether the null hypothesis of either test can or cannot be rejected. The test has four possible outcomes: equivalence if the difference test is non-significant and the equivalence test is significant, difference if the difference test is significant and the equivalence test is non-significant, trivial difference if the difference test is significant and the equivalence test is significant and indeterminacy if the difference test or the equivalence test are significant	‘to what extent does the effect size from the replication study differ or is equivalent to that of the original study?’	one original study and one replication study	to classify	first mentioned in Steiner *et al*. [77]
credibility analysis (reverse-Bayes, probability of credibility, probability of replicating an effect)	the analysis of credibility uses the results of a study (specifically the confidence interval) and uses a reverse-Bayes approach to find the prior that is required to generate credible evidence for the existence of an effect (i.e. a posterior that excludes no effect). The prior is then compared with internal or external evidence to assess if the finding is credible or not.	‘how credible are the results of a study, in a Bayesian framework?’	one original study	to quantify and classify	first mentioned in [78]. Discussed in [79,80]
cross-validation methods (jackknife, bootstrap)	internal cross-validation methodology are used to test result replicability, where the results received in one subsample of the raw data can be confirmed in the remaining data. The degree of shrinkage (validity shrinkage) is then estimated using the difference in $R^{2}$ between the subsamples, providing a theoretical basis to evaluate the reproducibility of result. The closer shrinkage is estimated to be zero, the greater the degree of stability and more confidence in the replicability/generalizability of the results. Alternatively, jackknife and bootstrap validation methods can be used	‘to what extent can the stability of a result be trusted, and to what extent can the result be generalized?’	one original study	to quantify and predict	first mentioned in [81]. Discussed in [82,83]
design analysis	given that a study was performed that yielded an estimate d with standard error s. Then a true effect-size D (the value that d would take if observed in a very large sample) has to be considered. The random variable $d^{rep}$ is defined as the estimate that would be observed in a hypothetical replication study with a design identical to that used in the original study. A probability model for $d^{rep}$ then gives the following three summaries: (i) the power: the probability that the replication $d^{rep}$ is larger (in absolute value) than the critical value that is considered to define ‘statistical significance’ in this analysis; (ii) the Type S error rate: the probability that the replicated estimate has the incorrect sign, if it is statistically significantly different from zero; and (iii) the exaggeration ratio (expected Type M error): the expectation of the absolute value of the estimate divided by the effect size, if statistically significantly different from zero	‘given the results of an original study and an effect of a hypothetical replication study, what is the probability of the estimate being in the wrong direction and what is the factor by which the magnitude of the effect is overestimated?’	one original study	to quantify and explain	first mentioned in [84]
difference in effect size (*Q*-statistic (meta-analytic), *Q*-test, difference test, Tukey’s post-hoc honest significant difference test)	the original and replication effect sizes can be compared by calculating their difference together with its confidence interval. They can further be compared in a significance testing paradigm using the Q-statistic or difference test. Alternatively, when there is data for several original-replication study pairs, a paired *t*‐test and/or Wilcoxon test can be applied on the effect size estimates for the original and replication studies. Tukey’s post-hoc honest significant difference test can be used to answer the question of how many replications produced results that were statistically indistinguishable from one another	‘to which degree do the effects from a replication study mirror the original?’	one original and one replication study; or several replications (meta-analytic Q-test)	to quantify and classify	first mentioned in [85] (Q-statistic for reproducibility). Discussed in [63,69,70,73,76,77,86–89]. Used in [15,17,24,29,30,32–34,37–40,42–44,46,55,64,90–101]
equivalence testing (two one-sided tests (TOST)	an equivalence range is constructed based on an equivalence margin, or a smallest effect size of interest. When assessing the replication of an original ‘null’ (non-significant) finding a successful replication would reject the null hypothesis of an effect being outside the equivalence region. Alternatively, when interested in assessing whether the original and the replication study find consistent or equivalent effects, one can test whether the difference in effect size falls within a region of equivalence	‘for the replication of an original null finding, does the replication study find an effect that is equally negligible?’—‘are the results from the replication statistically equivalent to the results of the original study?’	one original and one replication study	to quantify and classify	discussed in [13,63,77,89,102]
externally standardized residuals	for each i=1,…,n, the replication effect size i is compared to the weighted mean effect size of all replications, excluding study i via a standardized difference. These residuals can then inform on a failure to replicate. They tend to be ambiguous about successful replications. This metric is related to the measure of reproducibility of the studies included in a meta-analysis introduced by [103]	‘is the original study consistent with the replication(s)?’—‘are all studies included in a meta-analysis replicable?’	one original study and one replication; or one original study and many replications	to quantify and classify	first mentioned in [87]. Discussed in [103]
fragility index (fragility quotient)	the fragility index was proposed to quantify the robustness of the statistical significance of clinical studies with binary outcomes. It is defined as the minimal event status modifications that can alter statistical significance. If the original study result is statistically significant (with p(0,0)<α), the fragility index (FI) is defined as $$FI=\min_{p(f_{0},f_{1})\geq \alpha }|f_{0}|+|f_{1}|,$$ where $f_{0}$ and $f_{1}$ are the numbers of non-events changed to events in groups 0 and 1, respectively. If the original study result is non-significant (with p(0,0)≥α), the min is searched for all $f_{0}$ and $f_{1}$ with $p(f_{0},f_{1})<\alpha$. A smaller value of FI indicates a more fragile result. The FI was extended to meta-analyses and network meta-analyses. One may use the relative measure, fragility quotient (FQ), to compare the multiple studies’ fragility. Specifically: $FQ=\frac{FI}{n_{0}+n_{1}}\times 100\%,$ where $n_{0}+n_{1}$ is the total sample size of the study. Thus, the FQ represents the minimal percentage change of event status among all participants that can alter the significance (or non-significance), and it ranges within 0 and 10%	‘given the results of an original study were significant, what is the smallest change in the original data that is needed to deem the results non-significant? and vice-versa for original null results’—‘how fragile are the original results to small changes in the underlying data?’	one original study	to quantify	first mentioned in [104]. Discussed in [105,106]
I squared, $I^{2}$ (estimation of effect variance)	I squared (*I*^2^) describes the percentage of total variation across studies (replications) that is owing to heterogeneity rather than chance, and is calculated from basic results obtained from a typical meta-analysis: $$I^{2}=100\%\times (Q-\text{d.f.})/Q,$$ where Q is Cochran’s heterogeneity statistic and d.f. is the degrees of freedom. Any negative values of $I^{2}$ are set to zero so that it lies between 0 and 100%. A value of 0% indicates no observed heterogeneity, and larger values show increasing heterogeneity	‘given a set of replications, to what extent is the total variation across study results due to heterogeneity?’—‘how consistent are the results across replications?’	several replications; one original and several replications	to quantify	first mentioned in [107]. Discussed in [88]. Used in [31,42]
Jaccard similarity coefficient (coefficient of similarity)	the per cent overlap of activation between two functional magnetic resonance imaging (fMRI) studies (j and l) is defined as $$w_{j,l}=\frac{V_{j,l}}{V_{j}+V_{l}-V_{j,l}},$$ where $V_{j}$ and $V_{l}$ are the number of voxels identified as activated in either experiment and $V_{j,l}$ is the number of voxels identified as activated in both experiments. Wang *et al*. [108] suggest using a measure that is closely related to the Jaccard coefficient to measure reproducibility in omics data analysis	‘by what extent do the results of two (or more) fMRI experiments overlap?’	one original study and one replication study; or several replications	to quantify	discussed in [108,109]. Used in [110]
leave-one-out error	a model is trained on all data without the ith data point, and tested on the ith data point. The leave-one-out error is then directly related to the average loss or error over all i	‘given a deep learning model, how generalizable are its results?’	one original study	to quantify and predict	discussed in [111]
likelihood-based approach for reproducibility (likelihood-ratio)	the design of the original study is used to derive an estimate of a theoretically interesting effect size, $d_{\text{tie}}$. A likelihood ratio is then calculated to contrast the match of two models to the data from the replication attempt: a model based on the derived $d_{\text{tie}}$, and a null model. More specifically, a null model assumes no effect and a replication model that assumes the effect is $d_{\text{tie}}$. The magnitude of the likelihood ratio describes the strength of the evidence in favour of one or the other model. Very large ratios in favour of $d_{\text{tie}}$ would be considered strong evidence for replication. Symmetrically, very large ratios in favour of the null model would be strong evidence against replication	‘given a theoretically interesting effect size derived from the original study, what is the evidence for or against replicating this effect?’	one original study and one replication study	to quantify and classify	first mentioned in [112]
mean relative effect size (percentage difference in effect size)	the mean relative effect size is defined as $\nu =\sum_{j=1}^{m}\frac{\theta_{2j}/\theta_{1j}}{m},$ where $\theta_{2j}$ and $\theta_{1j}$ are the effect sizes from either the original or the replication study and m is the number of findings that were replicated. This value is usually used to assess by how much the effect size changed from original to replication study. Alternatively, the percentage difference can be used	‘what is the average ratio of replication study effects to original study effects?’	several pairs of original and replication studies	to quantify	discussed in [76]. Used in [15,30,40,56,110,113]
meta-analysis	fixed-effect or random-effects meta-analyses can be used to combine the results from an original and a replication study, or from several replication studies. In the pairwise scenario, a replication is often considered successful if the results of the meta-analysis align with the results of the original study (significance and direction of effect). When several replications are conducted of the same phenomenon, meta-analysis methodology can be used to assess the reproducibility of the finding. To account for potential heterogeneity between studies, random-effects models are used	‘given an original-replication study pair, does the pooled effect align with that of the original study?’—‘given a set of replications, is the effect size reproducible across studies?’	one original and one replication study; or one original and many replication studies; or several replications	to quantify and classify	discussed in [18,63,69,70,86,89,114,115]. Used in [16,17,32,40,42,43,64,116]
minimum effect testing	based on the results of the original study, a minimal level of evidence required to support the original study is defined, as a range constituting the null hypothesis. A test is performed to see whether the replication effect size lies within the range ($H_{0}$) or outside ($H_{1}$)	‘is the replication effect size significantly different from a minimal effect size of interest, required to support the original study?’	one original and one replication study	to classify	discussed in [63]. Used in [30]
network comparison test (NCT)	this test was proposed to statistically evaluate the similarity of network models	‘given two network structures, how similar are they to each other?’	one original study and one replication study	to quantify and classify	discussed in [117,118]
*p* interval	the *p* interval, or prediction interval for *p*, is an interval with a specified chance (usually 80%) of including the p-value given by a replication	‘given the results of an original study, what is the range of p-values a replication (following the same design) would lie in with 80% probability?’	one original study	to quantify and predict	first mentioned in [119]
prediction interval: replication effect in original 95% prediction interval	using the findings (effect size and variation) of the original study, and the expected variation of the replication study (linked to its sample size), compute the 95% prediction interval. This can be used to predict the effect size of the replication study or, for a binary criterion of replication success, check whether the replication effect size is included in the prediction interval. Schauer & Hedges [70] further show how the metric based on the prediction interval is related to the *Q*-test	‘do the findings from the replication study align with a reasonable expectation, given the observed variation in the original study and replication study?’—‘are the replication estimates statistically consistent with the original estimates?’	original finding only; one original and one replication study; or one original and many replication studies	to quantify and classify	first mentioned in [36]. Discussed in [63,69,70,76]. Used in [16,17,32,32,35] checked original effect in 95% prediction interval of replications
proportion of population effects agreeing in direction with the original, ${\hat{P}}_{>0}$	this metric assesses the strength of evidence of the replication effect sizes going in the same direction as the original effect size, by estimating the proportion of population effects agreeing in direction with the original effect estimate. It can be generalized by ensuring that they do not only agree in direction but are also stronger than a chosen threshold	‘to what extent do the replication effect sizes agree with the sign found in the original study?’	one original study and several replication studies	to quantify	first mentioned in [72]. Discussed in [41,73]. Used in [40]
quantified reproducibility assessment (QRA)	the method is based on the concepts and definitions of metrology. For QRA, the precision of measurements done in replications across varying conditions is assessed	‘after performing multiple measurements of an object, what is the precision of the measured quantity obtained?’	one original study and many replication studies	to quantify and classify	first mentioned in [120]. Discussed in [121,122]
replication Bayes factor	the replication Bayes factor tests the proponent’s replication hypothesis $H_{r}:\theta ∼$ posterior distribution from original study versus the null hypothesis $H_{0}:\theta =0$ of a sceptic who has reason to doubt the presence of an effect: $$B_{r0}=\frac{f(Y_{r}\;|\;H_{r})}{f(Y_{r}\;|\;H_{0})},$$ where $f(Y_{r}|H_{i})$ is the marginal likelihood of the data under hypothesis $H_{i}$ with i∈{0,1}. The higher the $B_{r0}$ the more evidence for the replication hypothesis.	‘what is the evidence for the effect from the replication attempt being comparable to what was found in the original study, or absent?’—‘are the replication results more consistent with the original study or with a null effect?’	one original and one replication study	to quantify	first mentioned in [60]. Discussed in [18,63,112,123,124]. Used in [64]
sceptical p-value (versions: nominal sceptical p-value, golden sceptical p-value, controlled sceptical p-value)	replication success is declared if the replication study is in conflict with a sceptical prior that would make the original study non-significant. The sceptical p-value quantifies the prior-data conflict. Held [125] introduced the nominal p-value. Two more recalibrations have been proposed since. The nominal p-value might be too stringent as it needs both original and replication study to be significant at level α. With the golden recalibration, it is possible to establish replication success, original and replication study do not both necessarily need to be significant at level α, provided that the replication effect estimate does not shrink compared to the original one. The controlled p-value was introduced to guarantee overall type I error control at $\alpha^{2}$ and is closely related to the significance criterion	‘to what extent are the results of a replication study in conflict with the beliefs of a sceptic of the original study?’	one original study and one replication study	to quantify and classify	first mentioned in [125]. Discussed in [18,126,127]
sceptical Bayes factor (reverse-Bayes)	the sceptical Bayes factor combines reverse-Bayes analysis with Bayesian hypothesis testing. First, a sceptical prior is determined for the effect size such that the original finding is no longer convincing in terms of Bayes factors. Then, this prior is contrasted to an advocacy prior (the reference posterior of the effect size based on the original study). Replication success is flagged if the replication data favour the advocacy over the sceptical prior at a higher level than the original data favoured the sceptical prior over the null hypothesis. The highest level for which replication success would be declared is then the sceptical Bayes factor	‘in light of the replication data, at which level of evidence can an advocate of the original study convince a sceptic?’	one original study and one replication study	to quantify and classify	first mentioned in [128]
significance criterion (vote counting, two-trials rule, regulatory agreement)	for an original-replication study pair, replication success is concluded when both original study and replication study find a statistically significant effect, in the same direction. This can be done either with directional two-sided hypothesis tests, or via a one-sided test. For a continuous assessment of reproducibility, $\max(p_{o},p_{r})$ can be used, where $p_{o}$ and $p_{r}$ are the p-values from the original and replication, respectively	‘do the original and replication study both find a statistically significant effect in the same direction?’	one original and one replication study; or several original-replication study pairs, or several replications	to quantify and classify	discussed in [13,18,63,69,70,76,77,89]. Used in [15–17,23,24,31–35,37–39,45–48,55,64,90–94,96,98,100,101,113,129,130]
small telescopes	based on the sample size and the statistical test performed in the original study, the effect that the original study has 33% power to detect, $d_{33}$, is computed. If the effect size of the replication study is significantly different from $d_{33}$, a replication failure is concluded	‘are the replication results consistent with an effect size big enough to have been detectable in the original study?’	one original and one replication study	to quantify and classify	first mentioned in [131]. Discussed in [18,63,112,123,132]
snapshot hybrid (Bayesian meta-analysis)	the method combines both the original and replication effect size to evaluate the common true effect size. It is a hybrid method because it only takes the statistical significance of the original study into account, whereas it considers evidence of the replication study as unbiased. The snapshot hybrid consists of three steps. First, the likelihood of the effect sizes of the original study and replication is calculated conditional on four hypothesized effect sizes (zero, small, medium and large). Second, the posterior model probabilities of these four effect sizes are calculated using the likelihoods of step 1 and assuming equal prior model probabilities. Equal prior model probabilities are selected by default, because this refers to an uninformative prior distribution for the encompassing model. Third, when desired, the posterior model probabilities can be recalculated for other than equal prior model probabilities	‘after replicating an original study, what is the evidence for a null, small, medium or large effect?’	one original study and one replication study	to quantify	first mentioned in [133]
Z-curve (exact replication rate, p-curves)	the Z-curve methodology is a method for estimating the expected replication rate, which can be defined as the predicted success rate of exact replication studies based on the mean power after selection for significance. An extension was proposed that estimates the expected discovery rate, in addition, which is the estimate of a proportion that the reported statistically significant results constitute from all conducted statistical tests and can be used to detect and quantify the amount of selection bias	‘do all studies combined provide credible evidence for a phenomenon?’	several replications or originals	to quantify and predict	first mentioned in [134] (Z-curve) [135], (P-curve). Discussed in [136]
**a framework**					
causal replication framework	the framework formalizes the conditions under which replication success can be expected, and allows for the causal interpretation of replication failures. These conditions are summarized into replication assumptions, which are qualitatively or narratively assessed. Replication failure occurs when one or more of the causal replication framework assumptions are violated	‘how can a replication failure be interpreted, from a causal perspective’	one original and one replication study; or one original and many replication studies; or several replications	to quantify and explain	first mentioned in [137]. Discussed in [138]
RepeAT (repeatability assessment tool)	the tool was developed using a multi-phase method to determine components needed for reproducing biomedical data: a literature review generated a framework, which was tested and refined. The RepeAT framework now contains 119 unique variables that were grouped into five categories, which address different components for reproducible research: research design and aim, database and data collection methods, data mining and data cleaning, data analysis, data sharing and documentation	‘does the presented research align with community standards of reproducible biomedical research, using electronic health records?’	one original study	to quantify	first mentioned in [139]
unified framework for estimating the credibility of published research	the unified framework for estimating the credibility of published research examines four fundamental falsifiability-related dimensions: transparency of the methods and data, reproducibility of the results when the same data-processing and analytic decisions are reapplied, robustness of the results to different data-processing and analytic decisions and reproducibility of the effect. This framework includes a standardized workflow in which the degree to which a finding has survived scrutiny is quantified along these four dimensions. More specifically, for method and data transparency: availability of design details, analytic choices and underlying data; for analytic reproducibility: ability of reported results to be reproduced by repeating the same data processing and statistical analyses on the original data; for analytic robustness: robustness of results to different data-processing and data-analytic decisions; and for effect reproducibility: ability of the effect to be consistently observed in new samples, at a magnitude similar to that originally reported, when methodologies and conditions similar to those of the original study are used. The framework outlines the steps to investigate these four dimensions	‘for a specific published research work, what is the evidence for its credibility measured on four different dimensions: method and data transparency, analytic reproducibility, analytic robustness and effect reproducibility?’	one original study and many replication studies	to quantify and explain	first mentioned in [140]
**a graph**					
Bland–Altman plot (agreement measures)	when two measures are compared (for example, replications and their original studies), the mean difference between the measures and standard deviations of the difference are used to define the limits of agreement. Then the average effect (average of replication and original effect) is plotted against the difference in effect size. The two measures can be used interchangeably if most of the points lie inside the limits of agreement. Other related agreement parameters can be used as well	‘do the effects estimated in several original-replication study pairs agree with each other?’—‘how good is the agreement between repeated measures/studies?’	several pairs of original and replication studies	to quantify and classify	first mentioned in [141]. Discussed in [142]. Used in [29,30]
modified Brinley plot	the plot summarizes the results for several replications, including a comparison (A versus B) by plotting the means of one phase (A, baseline) against the mean of the second phase (B, intervention) for each comparison. An identity line (diagonal with intercept = 0, slope = 1) is included to represent the lack of difference between means. A desired postintervention level and a desired amount of change after introducing the intervention is specified to define an area of the plot in which the dots should fall if they all meet both requirements. The share of points in the area gives the degree of replication	‘given a pre-specified desired effect and multiple replications, what is the share of replications that, represented graphically, achieve the desired effect?’	several replications	to quantify and classify	first mentioned in [143]. Discussed in [144]
reproducibility maps	the fMRI images are coloured depending on whether or not the truly active voxels were strongly reproducible or not	‘for fMRI research, how many and which of the truly active voxels were strongly reproduced?’	several replications	to quantify and classify	first mentioned in [145]
**a study, survey or questionnaire**					
prediction market	based on original results and information on the design of planned replication studies, participants in a prediction market trade contracts on the possible outcome of a replication study. The contracts pay a certain amount of money if the replication is successful. The traded contracts then allow the price to be interpreted as the predicted probability of the outcome occurring	‘what do the participants in a prediction market predict as the probability that the original findings will replicate?’	one original study with a planned replication; or several original studies with planned replications	to quantify and classify	first mentioned in [146]. Used in [16,48]
presence/absence of elements ensuring reproducibility, via proxies (framework for evaluating rigor and reproducibility)	an original paper is checked for the presence or absence of certain design and reporting elements that are crucial for its reproducibility. This is often achieved using checklists or reporting guidelines which summarize the community standards. The elements of these checklists or guidelines are usually integrated in a study, survey or questionnaire	‘do the design, methods and reporting of the original paper align with community standards of reproducible and transparent research?’	one original study	to quantify and classify	discussed in [121,147–149]
RepliCATS	the process elicits expert predictions about the reproducibility of research. It is based on a modified Delphi technique and includes four steps represented in the acronym IDEA: ‘Investigate’, ‘Discuss’, ‘Estimate’ and ‘Aggregate’. Each individual is provided a scientific claim and the original research paper to read, and provide an estimate of whether or not the claim will replicate (Investigate). They then see the group’s judgments and reasoning, and can interrogate these (Discuss). Following this, each individual provides a second private assessment (Estimate). A mathematical aggregation of the individual estimates is taken as the final assessment (Aggregate)	‘how reliable do experts believe the claims from an original finding are?’	one original study	to quantify and predict	first mentioned in [50]. Used in Alipourfard *et al*. [49]
subjective reproducibility assessment (replication standard, assessment of feasibility)	the replication teams are surveyed/asked to answer the question ‘Did your results replicate the original effect?’. The teams can give a binary answer, or give a more nuanced interpretation on, for example, a Likert scale. Specific fields have specified their own categories for reproducibility assessment, as, for example, the replication standard in agent-based modelling: ‘numerical identity’, ‘distributional equivalence’ and ‘relational alignment’. For the reproducibility of simulation studies, agreement between results from the replication studies and the original studies was assessed in a qualitative manner and involved evaluating: whether numerical values from the replication studies were comparable to those in the original studies, whether trends in the results were moving in the same direction, and whether the performance rankings of different simulation scenarios matched those in the original studies Luijken *et al*. [52]	‘does the replication team consider the replication as successful?’—‘to what extent does the replication team trust in the reproducibility of a finding?’	one original study and one replication study	to quantify and classify	discussed in [52,69,150]. Used in [15,23,29,34,35,45–48,51,52,55,57,100,151]
**an algorithm**					
reproducibility scale of workflow execution—Tonkaz	the metric is based on the idea of evaluating the reproducibility of results using biological feature values (e.g. number of reads, mapping rate and variant frequency) representing their biological interpretation. The resulting reproducibility scale is a 4- point scale and goes from ‘fully reproduced’ to ‘acceptable differences’ to ‘unacceptable differences’ to ‘not reproduced’. The authors implemented an automated system to classify results on this scale	‘given a certain original research paper with results based on computation, can the workflow to generate the results be executed and verified?’	one original study	to classify	first mentioned in [152]
RipetaScore	the ripetaScore combines three aspects of trust for a total of 30 points: (i) using the ‘Trust in Research’ criteria, it is determined whether a paper is a research paper. Only then will the paper continue to be scored; (ii) the paper is then evaluated for the presence of reproducibility quality indicators, and it can receive up to 20 points; (iii) another 10 points come from the trust in professionalism quality indicators. For the trust in reproducibility criteria, papers are primarily evaluated with regard to their data/code sharing practices, reporting of methods, and citing software. These criteria are all assessed via natural language processing	‘given certain trust in research, reproducibility and professionalism quality indicators, how high does a paper score?’	one original study	to quantify	first mentioned in [153]
text-based machine learning model to estimate reproducibility	a machine learning model using an ensemble of random forest and logistic regression was trained on data from replication studies. This model can then use a paper’s text and meta-data to predict its likelihood of replication, based on the significance criterion	‘given the text of an original paper, what is the probability of replication success?’	one original study; or several original studies	to quantify and predict	first mentioned in [154]. Discussed in [121,155]. Used in [49]

#### 3.4.3. Metrics based on formulas and statistical models

Of the identified metrics, 37 (37 out of 50 = 74%) were classified as being based on a formula or statistical model, making the majority ‘statistical metrics’. These metrics typically provide a quantitative assessment of reproducibility, with one exception: the correspondence test. This test, recently introduced by Steiner *et al*. [77], combines both difference and equivalence testing. While the two individual tests, which are also part of the identified metrics, provide a quantitative assessment, the correspondence test categorizes their combined outcome into four levels. At a pre-defined significance threshold α, it returns *equivalence* when the difference test finds no significant difference between the effect sizes of two studies and the equivalence test is significant. Alternatively, it can establish *difference*, *trivial difference* or *indeterminacy*. This test is particularly relevant when comparing an original study to its replication, addressing the question ‘to what extent does the effect size from the replication study differ or is equivalent to that of the original study?’. By contrast, the individual underlying tests provide more direct measures of the strength of evidence in terms of p-values.

The difference test, often referred to as *Q*-test (see ‘difference in effect size’ in table 4), has been widely used in large-scale replication projects, in some form or another. In a pairwise comparison of an original study with its replication, the research question addressed by this metric is ‘to which degree do the effects from a replication study mirror the original?’, which can be extended to ‘to which degree do the effects from a set of replication studies mirror each other?’ in a scenario where several replications are considered. This metric enables a direct comparison of effect sizes between two or more studies.

Most metrics that provide a quantitative assessment of reproducibility, can be dichotomized to classify a study as ‘reproducible’ versus ‘not reproducible’. We illustrate this using one of the most commonly used metrics for reproducibility: the significance criterion. When comparing two studies, the criterion deems the replication of an original study successful if both studies report a significant effect in the same direction at a pre-defined level α. This creates a binary outcome of either replication success or failure. To quantify the strength of evidence that both studies found a statistically significant effect in the same direction, the maximum p-value, $\max\{p_{o},p_{r}\}$, can be used, where $p_{o}$ and $p_{r}$ are the p-values from the original and replication. The binary classification is determined by checking whether $\max\{p_{o},p_{r}\}<\alpha$. This illustrates the scenario of pairwise comparisons between an original study and its replication. However, the same criterion was used in ‘many phenomena, one study’ projects, which involve multiple original-replication study pairs. In these cases, overall reproducibility was quantified by calculating the proportion of study pairs that achieve success [15,17,39]. Conversely, in projects of the type ‘one phenomenon, many studies’, where multiple replications test the same hypothesis or analyse the same data, reproducibility was quantified by determining the proportion of replications that yield statistically significant outcomes in the same direction [33,48,55]. This also shows how the same metric can be used to assess reproducibility in different contexts, such as when different methods are applied to the same dataset (e.g. ‘one phenomenon, many studies’), but also when the same methods are applied on different data (e.g. ‘many phenomena, one study’). Related to this, the metric called *P interval* offers a more nuanced interpretation of the p-value of an original finding by computing a prediction interval for the p-value of a hypothetical replication study [119]. Many included methodological review papers discuss the limitations of the significance criterion. For example, the significance criterion could potentially indicate replication failure even when the effect estimates in the original and replication study are the same. This is why some authors have designed metrics that combine the comparison of effect size with an investigation of the strength of evidence in the original and replication studies (e.g. the sceptical p-value [125,127] and the small telescopes approach [131]).

In addition to frequentist approaches for the assessment of reproducibility, some identified metrics included Bayesian methodology. For example, we identified Bayes factors (BFs) specifically designed for pairwise comparisons of original and replication studies: the equality-of-effect size BF [59], the replication BF [60] and the sceptical BF [128]. These Bayesian metrics assume probability distributions for the effect size parameters, which characterize epistemic uncertainty, while frequentist metrics assume unknown but fixed effects and are based on repeated sampling characteristics. Some of the identified metrics were designed to quantify reproducibility in a specific field of research, including the quantified reproducibility assessment developed for studies in natural language processing and the Jaccard similarity coefficient applied to functional magnetic resonance imaging (fMRI) research.

#### 3.4.4. Other types of metrics

We identified three metrics classified as *frameworks*. While we did not pre-define what a framework entails, these three were initially classified as ‘other’ but later grouped as frameworks, as this is how the authors described them. Frameworks generally present concepts in a structured way to help interpreting observations or results. The three frameworks we identified outline how various aspects of reproducibility can be combined into a more nuanced assessment. For example, the unified framework for estimating the credibility of published research evaluates aspects such as transparency of methods and data, computational reproducibility, robustness and effect reproducibility [140]. While it does not offer a final summary across these aspects, it collects diverse evidence for a nuanced qualitative judgment on reproducibility. The framework by McIntosh *et al*. [139], targeted at biomedical research, includes 119 items operationalizing research transparency that are integrated in an assessment tool (RepeAT). The iRISE glossary refers to such items as proxy measures [5]. Although the authors suggest automation, its implementation remains unclear. Unlike the latter frameworks, which are useful to quantify or assess the reproducibility of one or several original studies, the causal replication framework by Steiner *et al*. [137], is designed for use when at least one replication study is available or planned. It helps interpret and explain replication outcomes by examining the assumptions under which replication success can be expected. These frameworks are different from other metrics listed above as they tend to give more nuanced conclusions and interpretations specific to different contexts, instead of an overall reproducibility quantification.

Among the graphical representations identified, Bland–Altman plots have long been used in medical research to assess the agreement of two measurements. Wang *et al*. [30] used this plot to assess the computational reproducibility in real-world evidence studies, while Page *et al*. [29] used it for agreement between original and replication effect sizes in evidence synthesis. These examples highlight the plot’s potential applications to different aspects of reproducibility: computational reproducibility in the first case and conceptual replication in the second, as defined in Voelkl *et al*. [5]. Other graphical representations, such as *reproducibility maps* (specific for fMRI research) and modified Brinley plots (more broadly applicable to a setting of several replications of the same intervention study), were developed specifically for reproducibility.

Four identified metrics involved actual studies, where participants, often field experts, assess the reproducibility of studies. The participants in prediction markets, used in two of our application papers [16,48], trade contracts which will be worth a certain amount of money based on replication outcomes. The final price of the contracts will reflect the predicted probability of successful replication. Prediction markets are most applicable when a set of original studies are planned to be replicated. Other metrics use survey techniques to evaluate whether an original study’s design, methods or reporting meet community standards of reproducible research. The RepliCATS methodology uses a modified Delphi process, where experts are asked to reach a consensus on the reproducibility of a study in several rounds before the data are aggregated into a final reproducibility assessment. In many ‘many phenomena, one study’ projects, replication teams are asked to assess replication outcomes using a binary scale (success/failure) or a more nuanced scale (e.g. Likert). While the implementation of these metrics was generally classified as clear, they can be labour- and cost-intensive because of the need to recruit participants, or pay participants in prediction markets.

Finally, we identified three algorithm-based metrics. Two involve checking the presence or absence of certain reproducibility-related proxy features using automated software tools. Another algorithm uses machine learning models to quantify reproducibility based on the texts and meta-data of a study. These algorithm-based metrics are useful for evaluating the reproducibility of single original studies, but might again come with substantial costs, as they are computationally extensive, or require specialized software and IT knowledge.

### 3.5. Equity, diversity and inclusion considerations in reproducibility assessment

For 18 of the 49 application papers (37%) and 15 of the 95 methodological papers (16%), we extracted content related to EDI. The extracted text was grouped into five themes: *diversity in replication teams*, *diversity in replication samples*, *epistemic diversity*, *generalization of findings* and *research culture*. Methodological papers overwhelmingly focused on epistemic diversity, defined as the diversity of knowledge production, expertise, field, method of study, epistemic values and/or reasoning [5,156]. This epistemic diversity was reflected in the methods papers either via encouraging future studies generalizing the metric to fields other than those initially proposed, or an explanation that the metric is only relevant for a specific field or method of study. Application papers were more likely to encourage diversity of replication teams (those conducting replication studies) or replication samples (both human and non-human samples). Several application papers highlighted the importance of generalizability and heterogeneity, noting that increased diversity and heterogeneity in replications may lead to increased generalizability when findings of multiple replications are considered in aggregate. Finally, two papers (one application and one methodological) noted the relevance of research culture to reproducibility and reproducibility metrics, suggesting that social and cultural factors can facilitate or impede uptake of reproducible research practices and replication projects. The raw data containing the extracted texts on EDI considerations are available via osf.io/sbcy3/.

## 4. Discussion

In this study, we systematically searched the methodological literature on metrics to quantify, assess, explain, or predict reproducibility. This review was complemented by an investigation into the reproducibility metrics that have so far been used in large-scale replication projects. Our search included 49 replication projects and 95 distinct methodological papers. We identified 50 different metrics and summarized them in a table which organized the metrics by type—formulas or statistical models, frameworks, graphs, studies, surveys or questionnaires, and algorithms. When conceptualizing this review, we did not expect to find such a high number of metrics. The fact that they are diverse in nature and address slightly different questions and aspects of reproducibility, underpins the complexity of measuring reproducibility. Therefore, there cannot be a single, universally applicable reproducibility metric; it should be a case-by-case choice aligned with the goals of the study.

Classifying the metrics to one specific type of reproducibility was not straightforward and might not even be possible. While many metrics have been developed or applied with one aspect of reproducibility in mind, they can often be directly applied or can be extended to other aspects. Future research focusing on specific aspects of reproducibility can build on our results by selecting the metrics to apply in that context and investigate their assumption and limitations. Our reproducibility metrics table is an important contribution that provides a clear overview of available metrics, their potential applications and references for further information. We hope that it will serve as a practical tool for future replication teams to plan their projects more effectively, as it offers a way to align the type and aim of a study with the most appropriate metric(s), based on the research questions under consideration. The metrics table additionally offers opportunities for researchers to explore new metrics and make informed decisions on which metrics best fit their study design, and constraints. For those new to the field, considerations related to cost and ease of implementation of the various metrics are highlighted in the online version of our table (http://rachelhey.github.io/reproducibility_metrics/). Peer-reviewers can use the table to critically review reproducibility studies regarding the appropriateness of the metric(s) used. Meta-researchers can find reproducibility outcomes for future intervention studies aiming at improving reproducibility. Our table can help to align reproducibility metrics to the goals of a replication effort [13] or reproducibility studies. Researchers who want to follow the recommendation that the design of replication efforts should be informed by the reproducibility metrics [157], may find the information in the table helpful. A noteworthy observation from our data extraction is that large-scale replication projects rarely provide a definition of reproducibility. Additionally, while these studies put a lot of effort into describing the design and methods used in the replication, they seldom outline the methods used to summarize reproducibility. Instead, they tend to only report the results in a descriptive manner in the results section. Therefore, we invite researchers to choose the metric(s) that align(s) with their research question and justify this choice. Sharing data and code could further allow for the assessment of the performance of other metrics or how they interact and complement each other in practice.

In an exploratory analysis, we extracted any mention of EDI dimensions. As expected, only a handful of papers included such considerations, but we could still find some valuable data which will be useful in the remainder of the iRISE project, which includes a work package examining the interface of reproducibility and research culture. Our study also shows, however, that EDI dimensions are explicitly considered only in few instances, and should be given higher priority in future work.

### 4.1. Limitations

While our search strategy was extensive, we cannot be sure that the list of metrics is fully exhaustive. Owing to the epistemic diversity in the understanding of reproducibility, it is possible that we missed relevant metrics because our keywords did not capture this diversity. Other metrics or tools that measure reproducibility-related proxies, including reporting or transparency as for example SciScore [158,159], might not have been identified in our review as they have not been presented as metrics assessing or quantifying reproducibility, even though they could be used as outcome measures for future reproducibility studies. Additionally, our review only captures a snapshot in time, and we hope to update the online, ‘live’ version of our table whenever new metrics become available (as, for example, Held *et al*. [160], which was published after our literature search). Therefore, the research community is invited to suggest the addition of other reproducibility metrics (by contacting the corresponding author or by creating an issue on our GitHub page). Second, we did not critically evaluate or scrutinize the quality or effectiveness of the metrics identified, but rather focused on collecting and characterizing them. Future research should build on this work and involve a rigorous assessment of the metrics to better understand their strengths and weaknesses. Third, specifically for the application papers, we did not investigate the relationship between the metrics used and the outcome of the projects. For instance, different metrics might produce conflicting results, where one indicates replication success or high reproducibility while the other suggests failure or low reproducibility. Finally, owing to resource constraints, we decided to exclude single-study application papers from our review. While they, as described above, generally use the same set of metrics, it could be that the way results are analysed differs from large-scale studies (e.g. because researchers can zoom in closer, as there is only one original-replication pair). This could be another avenue for future research and complement our review of large-scale replication projects, as well as the work done by Cobey *et al*. [14].

## 5. Conclusion

Our review offers a comprehensive overview of various reproducibility metrics. By providing classifications of their types, their potential applications and ease of implementation, we hope to assist future replication teams and meta-researchers to make informed research decisions. We have also paved the way for future research to critically evaluate these metrics further and explore real-world implications.

## Data Availability

All records included (after screening) in our review are organized in a Zotero library (https://www.zotero.org/groups/5397531/reproducibilitymetrics), and the methodological papers from the literature search are included in another Zotero library (https://www.zotero.org/groups/5630395/reproducibilitymetrics_methodsscreening/library). The complete set of records screened for the methodological papers is available via https://www.zotero.org/groups/5630395/reproducibilitymetrics_methodsscreening. Data files with the data extraction of both application and methodological papers are stored on the Open Science Framework (OSF) [161]. Relevant code to produce summary statistics, figures and tables are stored in GitHub: https://github.com/rachelHey/reproducibility_metrics and has been linked within our OSF page.

## Footnotes

^1^
We define a reproducibility study as any type of study investigating the reproducibility of a field, study, analysis or finding.

^2^
These were defined as larger projects where a group or a consortium of researchers attempt to reproduce a set of original studies, or the same original study several times, in order to make claims about the reproducibility of a field of study or type of research.

^3^
https://www.zotero.org/groups/5397531/reproducibilitymetrics/collections/HST4PWW8.

## Appendix A. Search strings for methodological papers

### A.1. Scopus

TITLE-ABS((replication* OR replicated OR reproduced OR reproduction* OR generalised OR generalisation* OR generalized OR generalization) W/1 (study* OR studies OR experiment* OR analys* OR analyz* OR estimation* OR estimate* OR result* OR finding*) OR ((reproducibility W/2 research) OR (reproducibility W/2 science) OR (replicability W/2 research) OR (replicability W/2 science) OR (generalisability W/2 research) OR (generalisability W/2 science) OR (generalizability W/2 research) OR (generalizability W/2 science) OR (translatability W/2 research) OR (translatability W/2 science))) AND TITLE-ABS ((replicable OR replication OR replicability OR reproduction OR reproducible OR reproducibility OR generalisable OR generalisability OR generalisation OR generalizable OR generalizability OR generalization OR translatable OR translation OR translatability) W/1 (quantif* OR measure* OR metric* OR evaluat* OR score* OR assess* OR rating* OR ratio* OR rate*)) AND (LIMIT-TO (DOCTYPE,‘ar’) OR LIMIT-TO (DOCTYPE,‘cp’))

### A.2. Ebsco

((((TI replication* OR AB replication*) OR (TI replicated OR AB replicated) OR (TI reproduction* OR AB reproduction*) OR (TI reproduced OR AB reproduced) OR (TI generalisation* OR AB generalisation*) OR (TI generalised OR AB generalised) OR (TI generalization* OR AB generalization*) OR (TI generalized OR AB generalized)) N1 ((TI study* OR AB study*) OR (TI studies OR AB studies) OR (TI experiment* OR AB experiment*) OR (TI analys* OR AB analys*) OR (TI analyz* OR AB analyz*) OR (TI estimation* OR AB estimation*) OR (TI estimate* OR AB estimate*) OR (TI result* OR AB result*) OR (TI finding* OR AB finding*))) OR (((TI reproducibility OR AB reproducibility) N2 (TI research OR AB research)) OR ((TI reproducibility OR AB reproducibility) N2 (TI science OR AB science)) OR ((TI replicability OR AB replicability) N2 (TI research OR AB research)) OR ((TI replicability OR AB replicability) N2 (TI science OR AB science)) OR ((TI generalisability OR AB generalisability) N2 (TI research OR AB research)) OR ((TI generalisability OR AB generalisability) N2 (TI science OR AB science)) OR ((TI generalizability OR AB generalizability) N2 (TI research OR AB research)) OR ((TI generalizability OR AB generalizability) N2 (TI science OR AB science)) OR ((TI translatability OR AB translatability) N2 (TI research OR AB research)) OR ((TI translatability OR AB translatability) N2 (TI science OR AB science)))) AND (((TI replicable OR AB replicable) OR (TI replication OR AB replication) OR (TI replicability OR AB replicability) OR (TI reproduction OR AB reproduction) OR (TI reproducible OR AB reproducible) OR (TI reproducibility OR AB reproducibility) OR (TI generalisable OR AB generalisable) OR (TI generalisability OR AB generalisability) OR (TI generalisation OR AB generalisation) OR (TI generalizable OR AB generalizable) OR (TI generalizability OR AB generalizability) OR (TI generalization OR AB generalization) OR (TI translatable OR AB translatable) OR (TI translation OR AB translation) OR (TI translatability OR AB translatability)) N1 ((TI quantif* OR AB quantif*) OR (TI measure* OR AB measure*) OR (TI metric* OR AB metric*) OR (TI evaluat* OR AB evaluat*) OR (TI score* OR AB score*) OR (TI assess* OR AB assess*) OR (TI rating* OR AB rating*) OR (TI ratio* OR AB ratio*) OR (TI rate* OR AB rate*)))

## Appendix B. Screening guides for methodological papers

### B.1. First screening of search results for methodological papers

**What papers will be included?**

— Methodological papers—does title and abstract suggest that the paper presents/discusses a measure to quantify reproducibility?
Definition of ‘methodological papers’ in our setting (adapted from [162]) is any paper that
  — describes methods or measures to quantify the reproducibility of a field, a finding, an effect, a study, a method, …—example: [41]; and
  — describes methods or measures to assess a successful reproduction—example: [63].
This includes:
  — review papers, but only if the paper reviews methods or measures to quantify reproducibility or assess successful reproductions, e.g. ‘methodological review papers’—example: [11] [OPTION TO FLAG AS REVIEW PAPERS];
  — tutorial papers, explaining or demonstrating how to use measures to quantify reproducibility. [OPTION TO FLAG AS TUTORIAL PAPERS]; and
  — commentaries and editorials, if it is apparent from the abstract that a new alternative measure is suggested/discussed.
— Application papers are included only if it is apparent from the abstract that they use an innovative measure to quantify reproducibility [edge case—OPTION TO FLAG AS INTERESTING APPLICATION PAPER].
— Papers investigating any type of reproducibility can be investigated—we use reproducibility as an overarching term for aspects including computational reproducibility, replicability, translatability and generalisability. See the iRISE glossary for more definitions [5].
— Papers discussing reproducibility in any discipline or field of study are included.
— Papers published in any year are included (until 13 May 2024).

**What papers will be excluded?**

— Application papers—whenever it is clear from the title and abstract that the paper presents a reproducibility study (single study or large-scale project) and only applies a certain measure, we will exclude it.
— Review papers that are not ‘methodological review papers’, reviewing methods or measures to quantify reproducibility or assess successful reproductions, are excluded.
— Papers that are off topic (while using the same terminology), e.g. translation in linguistics, image replication, sexual reproduction, cell or bacteria replications or replicability, virus reproduction ratio, etc.
— Editorials are excluded if they are not discussing a new measure.
— Commentaries are excluded if they are not discussing a new measure.

### B.2. Second screening of list of potential methodological papers

**What papers will be included?**

— Methodological papers—does title and abstract suggest that the paper presents/discusses a measure to quantify, predict or explain reproducibility? This includes more quantitative measures of reproducibility but also qualitative investigations, e.g. Delphi studies.
Definition of ‘methodological papers’ in our setting (adapted from [162]) is any paper that:
  — describes methods or measures to quantify, predict or explain the reproducibility of a field, a finding, an effect, a study, a method, …—example: [41]; and
  — describes methods or measures to assess a successful reproduction—example: [63].
This includes
  — review papers, but only if the paper reviews methods or measures to quantify reproducibility or assess successful reproductions, e.g. ‘methodological review papers’—example: [11];
  — tutorial papers, explaining or demonstrating how to use measures to quantify reproducibility; and
  — commentaries and editorials, if it is apparent from the abstract that a new alternative measure is suggested/discussed.
— Application papers are included only if it is apparent from the abstract that they use an innovative measure to quantify reproducibility [edge case—OPTION TO FLAG AS INTERESTING APPLICATION PAPER].
— Papers investigating any type of reproducibility can be investigated—we use reproducibility as an overarching term for aspects including computational reproducibility, replicability, translatability and generalizability. See the iRISE glossary for more definitions [5].
— Papers discussing reproducibility in any discipline or field of study are included.
— Papers published in any year are included (until 13 May 2024).

**What papers will be excluded?**

— All types of application papers—whenever it is clear from the title and abstract that the paper presents a reproducibility study (single study or large-scale project) and only applies a certain measure, we will exclude it.
— Review papers that are not ‘methodological review papers’, reviewing methods or measures to quantify reproducibility or assess successful reproductions, are excluded.
— Papers that are off topic (while using the same terminology), e.g. translation in linguistics, image replication, sexual reproduction, cell or bacteria replications or replicability, virus reproduction ratio, etc.
— Editorials are excluded if they are not discussing a new measure.
— Commentaries are excluded if they are not discussing a new measure.

## Appendix C. Data extraction questions

### C.1. Guide for data extraction of *application papers*

(1) Field of research—as described by the authors. Select from list of the broader fields (select all that apply):
  (a) social sciences and humanities
  (b) life sciences
  (c) STEM, e.g. engineering, mathematics, physics
  (d) n/a
(2) Discipline—as described by the authors (add up to three disciplines, if more than three, write interdisciplinary).
(3) Type of project—one of the following:
  (a) many phenomena, one study (e.g. the Reproducibility Project Psychology): many original hypotheses are tested. Each hypothesis is tested in one (replication) study;
  (b) one phenomenon, many studies (e.g. multilab replication studies): one original hypothesis is tested by many different teams/in many separate studies;
  (c) many phenomena, many studies (e.g. FORRT Replications & Reversals, Replication Database, FORRT Replication Database): many original hypotheses are tested, each hypothesis is tested in many separate studies
  (d) other (add as comment).
(4) Did the authors define the type of reproducibility that is investigated?—yes or no:
  (a) (if yes—child question of above). Aspect of reproducibility investigated (authors)—extract definition of reproducibility reported by the authors.
(5) Even if defined by the authors, infer the aspect of reproducibility investigated using the concept of reproducibility as it is presented in *The Turing Way* matrix [18]. To this end, select one or several of the following:
  (a) same data—same analysis;
  (b) same data—different analysis;
  (c) different data—same analysis
  (d) different data—different analysis; and
  (e) other (add as comment).
(6) Did the authors measure reproducibility, i.e. summarize the results, using one of the following traditional measures (select all that apply and add details in comment cell):
  (a) agreement in statistical significance (add details in the comment cell)—example: are original and replication p-values <0.05?;
  (b) agreement in effect size (add details in the comment cell)—examples: do original and replication effect size go in the same direction? Is the replication effect size smaller than the original effect size? Is the replication effect size contained in the original effect size 95% confidence interval? Is the original effect size contained in the replication effect size 95% confidence interval? Is the replication effect size contained in a 95% prediction interval based on the original effect size?;
  (c) meta-analysis of original and replication study/studies (add details in the comment cell)—examples: is meta-analytic p-value <0.05? How large is meta-analytic effect size? Does meta-analytic 95% confidence interval include zero? Is there evidence for heterogeneity, e.g. p-value from *Q*-test <0.05?;
  (d) Subjective assessment (add details in the comment cell)—examples: answer of replicators to ‘did it replicate’?, Answer of original authors to ‘did it replicate’?; and
  (e) none of the above.
(7) Did the authors measure reproducibility, i.e. summarize the results, using one or several measures not present in the previous list? Add all:
  (a) (if yes—child question of above). Paste description of all other measures used.
(8) Did the paper refer to other papers for more information on the metric(s) used?—yes or no:
  (a) (if yes—child question of above). Paste the DOI of all paper(s) and add the name of the metric it refers to (as called in previous questions) in the comment.
(9) Did the authors discuss limitations or assumptions of the metric(s) used?—yes or no:
  (a) (if yes—child question of above). Paste text on limitation/assumptions and add the name of the metric it refers to (as called in previous questions) in the comment.
(10) Did the authors discuss equity, diversity, and/or inclusion (see definition below) at any point?—yes or no:
  (a) (if yes—child question of above). Paste text.
(11) Research question or aim—if obvious, paste research question or aim as reported by authors, for example, ‘to estimate the reproducibility of field XYZ’.

### C.2. Guide for data extraction of *methodological papers*

#### C.2.1. Interesting application papers

(1) Did the authors define the type of reproducibility that is investigated?—yes or no:
  (a) (if yes—child question of above). Aspect of reproducibility investigated (authors)—extract definition of reproducibility reported by the authors.
(2) Even if defined by the authors, infer the aspect of reproducibility investigated using the concept of reproducibility as it is presented in *The Turing Way* matrix [18]. To this end, select one or several of the following:
  (a) same data—same analysis;
  (b) same data—different analysis;
  (c) different data—same analysis;
  (d) different data—different analysis; and
  (e) other (add as comment).
(3) How did the authors measure reproducibility, i.e. summarize the results? Paste description of all measures used.
(4) Did the paper refer to other papers for more information on the metric(s) used?—yes or no:
  (a) (if yes—child question of above). Paste the DOI of all paper(s).

#### C.2.2. Methodological papers

(1) **Type of paper**
  *Detailed description*: what type of paper are you annotating?:
  — original research paper;
  — review paper—a review of measures/metrics to quantify reproducibility;
  — tutorial paper;
  — protocol—study protocol and alike, where the study might still be ongoing;
  — editorial, comment or similar; and
  — other (add a comment).
(2) **Design purpose**
  *Detailed description*: was (were) the presented measure(s) ‘designed’ for reproducibility? Some methods were developed for another purpose, but might have been used to quantify or assess reproducibility, in the application papers. yes, no, unclear (explain in comment).
(3) **Name of reproducibility (or related concept**)
  *Detailed description*: (free text) how did the authors ‘call’ what they are investigating? If they used more than one re-term, e.g. reproducibility and replication study, add all of them. Whenever possible, use the terms from the iRISE glossary. If unclear, or you cannot find a name, leave blank.
(4) **Definition of reproducibility (or related concept**)
  *Detailed description*: can you find a clear definition of the type of reproducibility, or related concept, the authors are interested in? An example of a clear definition would be: reproducibility is commonly defined as the ability to obtain ‘consistent results using the same input data, computational steps, methods and conditions of analysis’ (from 10.1016/j.cmpb.2023.107839). An unclear definition would be: direct replications of the original study, all following the same vetted protocol—as this text snippets only explains what has been done, but was not meant as a definition (from 10.1177/1745691616664694):
  — yes, clear definition (add text in comment);
  — yes, but unclear defined (add text in comment); and
  — no.
(5) **Type of reproducibility (or related concept**)
  *Detailed description*: (multiple choice) what is the type of reproducibility investigated using the discussed measure(s). Even if defined by the authors, infer the aspect of reproducibility investigated using the concept of reproducibility as it is presented in *The Turing Way* matrix [18]. Note that same data = using exactly the same data as the original authors, or the exact same data source and data retrieval steps; and same analysis = following a pre-defined set of steps, allowing for slight variations. To this end, select one or several of the following:
  — same data—same analysis;
  — same data—different analysis;
  — different data—same analysis;
  — different data—different analysis;
  — other (add as comment); and
  — unclear (explain in comment).
(6) **Purpose of measure**
  *Detailed description*: (multiple choice) what is (are) the measure(s) meant to be used for? Note that one measure can be used for several (or even all) of these purposes. Select all that apply and are discussed in the paper. Use the comment field to give context, or further explanation:
  — to quantify (continuous) reproducibility or related concept. Example: a method that estimate reproducibility rates/probabilities and alike—https://doi.org/10.15626/MP.2021.2720;
  — to classify (binary, yes or no) reproducibility or related concept. Example: a tool or similar that gives a yes-no answer to the question ‘is this reproducible’—https://doi.org/10.6084/m9.figshare.c.5418242;
  — to predict reproducibility or related concept. Example: a model/algorithm/tool that uses the findings or the text of one paper to predict how well the study or the findings would reproduce—https://www.pnas.org/doi/10.1073/pnas.1909046117;
  — to explain reproducibility or related concept. Example: a model which tries to explain certain levels of reproducibility with covariates and alike—https://doi.org/10.1111/insr.12273; and
  — unclear (explain in comment).
(7) **Number of measures**
  *Detailed description*: (free text) how many distinct measures, methods or models are discussed in the paper? Usually only one, but if the paper is a review, or if several variants of a method are discussed/presented, there might be more (example—this preprint discusses Edgington’s method and also presents a weighted version of the same methods). If the same measure is applied in different contexts or in various studies, the number of measures is still only one. Add a comment, if the number reflects the number of variants of the same measure/method.
(8) **Type of measure**
  *Detailed description*: (multiple choice) what type of measure(s) is (are) discussed? Note that some measures might use a combination of these types. Select all that apply, and explain in comment:
  — a formula, e.g. a percentage, a p-value, a Bayes factor;
  — a statistical model, e.g. a model which relates ‘reproducibility’ or a proxy thereof to some covariates;
  — an algorithm, e.g. a tool that uses unstructured data, like text, to estimate a reproducibility rate;
  — a study, e.g. a Delphi study is set up to assess the reproducibility of a study;
  — a survey or questionnaire, e.g. a set of experts are asked, via a survey, whether they rate a study as fully, partially or not at all reproducible;
  — other (explain in comment); and
  — unclear (explain in comment).
(9) **Type of assessment**
  *Detailed description*: (multiple choice) is (are) the measure(s) of quantitative or qualitative nature? A quantitative measure would give a continuous result, while a qualitative measure would rather give a classification into something like ‘fully reproducible’, ‘partially reproducible’, ‘not reproducible’. Some measures are deterministic and do not need any subjective input, others rely, at least in some way, on a subjective assessment. If the paper discusses several measures, with some being quantitative and others qualitative, select all that apply and add a comment:
  — quantitative;
  — qualitative;
  — objective;
  — subjective; and
  — unclear (explain in comment).
(10) **Name of measure**
  *Detailed description*: (free text) how did the authors call the measure? Leave blank if they did not name the measure. If they are discussing more than one measure, add all their names separately.
(11) **Implementation of measure**
  *Detailed description*: (multiple choice) we are interested in knowing whether the discussed measure(s) can be easily implemented by a researcher who wants to investigate reproducibility. Use the comment field to give more context. Select all that apply, especially if several measures are investigated:
  — ready-to-use open-source tool—the authors provide a tool, a code script or similar to use the suggested measure(s), at no added costs;
  — ready-to-use closed tool—the authors implemented or used a tool, a code script or similar to use the suggested measure(s), but it might come at a cost or the access is restricted;
  — easy to implement—the measure(s) discussed can be easily implemented, and the authors gave enough details to do so (e.g. using available software and instructions);
  — hard to implement—the measure(s) discussed can be implemented, but it is not straightforward, labour- or time-intensive (e.g. a Delphi study is implementable, but time-consuming) or expensive;
  — unclear implementation—the authors did not give enough detail on how to implement the measure
  — suggested only—the authors suggest a measure(s) or a general way to investigate reproducibility, but do not give guidance on how to actually use it; and
  — unclear (explain in comment).
(12) **Data input of measure**
  *Detailed description*: (multiple choice) when applying the measure to investigate reproducibility or related concept, what is the input of the measure, as in on what will the measure base its assessment on? Select all that apply. If more context or explanation is needed, use the comment field:
  — text;
  — some demographics or meta-data;
  — code or software;
  — results—numbers and tables;
  — results—figures;
  — qualitative data, surveys or questionnaires;
  — other (add in comment); and
  — unclear (explain in comment).
(13) **Assumptions or prerequisites for measure’s usage**
  *Detailed description*: (free text) to use the measure, does the input need to be in a certain form, follow a certain distribution or does the user need specific software and alike? Write down all assumptions and/or prerequisites (like needed software) that the authors mention. If you are writing down assumptions and prerequisites of specific measures, if possible, add the name of the measure you are referring to. Leave blank if the authors did not discuss anything.
(14) **Limitation of measure**
  *Detailed description*: (free text] did the authors discuss limitations of the measure(s)? If yes, write down all the discussed limitations you find (they might be referring back to prerequisites or assumptions—just write them down as limitations too). If you are writing down limitations of specific measures, if possible, add the name of the measure you are referring to. Leave blank if the authors did not discuss anything.
(15) **Equity, diversity and/or inclusion**
  *Detailed description*: (free text) did the authors discuss equity, diversity and/or inclusion (see definition below or the iRISE glossary—second part on EDI) related to the usage of the measure, at any point? Specifically, epistemic diversity (diversity of knowledge production, expertise, field of study, method of study, etc.) might be something that is discussed more often—e.g. if the measure is suggested in one specific field, can it be used in another, etc? Leave blank if the authors did not discuss anything.
